# Ornamental horticulture in Southern Africa: strategic actions to address biological invasions

**DOI:** 10.1007/s00267-025-02241-y

**Published:** 2025-08-28

**Authors:** Diana Rodríguez-Cala, Jana Fried, John R. U. Wilson, Katharina Dehnen-Schmutz, Seoleseng O. Tshwenyane, Israel Legwaila

**Affiliations:** 1 Centre for Agroecology, Water and Resilience, Coventry University, Coventry, UK; 2 Department of Crop and Soil Science, Botswana University of Agriculture and Natural Resources, Gaborone, Botswana; 3 Centre for Invasion Biology, Department of Botany and Zoology, Stellenbosch University, Stellenbosch, South Africa; 4 South African National Biodiversity Institute, Kirstenbosch Research Centre, Cape Town, South Africa

## Abstract

Southern Africa has a well-documented history of intentional plant introductions for ornamental purposes, but some of these plants have become widespread damaging invaders. Conflicts can arise when stakeholders’ attitudes differ towards ornamental invasive plants and their management. We examined the views of stakeholders involved in the ornamental sector and environmental management across Southern Africa in light of the strategic actions proposed by the Intergovernmental Science-Policy Platform on Biodiversity and Ecosystem Services' thematic assessment on ‘Invasive Alien Species and their Control.’ Our analysis is based on semi-structured interviews, informal conversations, and observations with 78 environmental specialists, 30 ornamental-related industry staff, and 24 plant enthusiasts from Botswana, Namibia, Zimbabwe, South Africa, Zambia, Democratic Republic of Congo, and Eswatini. Our analysis shows that significant efforts are ongoing in Southern Africa to address biological invasions from the ornamental sector. However, they need more integration and consideration of the broader geopolitical and socio-historical context. We reflected on these needs and recommend: 1) improving cohesion and collaboration amongst stakeholders, 2) ensuring pluralism by recognising and valuing marginalised groups, 3) addressing power differences and superiority-inferiority complexes, and 4) seeking alliances with existing sub-regional groups working in the realm of nature-society interplay. We believe that our recommendations contribute toward the necessary transformative change for tackling the underlying political and economic causes of plant invasions derived from the ornamental sector in the sub-region.

## Introduction

Globally, the ornamental horticulture sector has a long history of human-plant movement and, thus, of plant invasions (van Kleunen et al. 2018). In Southern Africa alien plants are widely traded and used as ornamentals (Mukubu Pika et al. 2024; Semenya and Maroyi 2020), and introductions for that purpose might even be on the rise (Faulkner et al. 2023). Several species introduced as ornamentals have become invasive (e.g. cacti, lantana) (Novoa et al. 2017; Vardien et al. 2012). Furthermore, 57% of the alien species that showed the highest range expansion in South Africa between 2000 and 2016 were introduced as ornamentals (Henderson and Wilson 2017). These estimates highlight the invasiveness of such plants and the ongoing long-lasting effects that introductions for ornamental purposes might have in the sub-region.

The management of some ornamental invasive plants, like cacti, has stirred conflicts in South Africa due to opposing attitudes between, for example, nursery owners and land managers (Novoa et al. 2016). Varying attitudes to invasive species management might primarily arise from stakeholders’ divergent interests, beliefs, and values, which, in turn, vary with broader socio-ecological (and geopolitical) contexts (Shackleton et al. 2019a). Invasive ornamental plants can be a livelihood, a hobby, and a liking for some people; while, at the same time, damaging livelihoods, lives, and the environment of others. Such perceptions can be highly dynamic over time and space (Shackleton et al. 2019a). Therefore, to effectively manage invasive species linked to the ornamental sector in Southern Africa, coordinated and systematic strategic actions that account for such dynamics need to be included. Furthermore, such strategic actions would require closer collaboration across the ornamental sector and neighbouring countries due to the transboundary nature of plant trade and biological invasions (Faulkner et al. 2017; Rodríguez-Cala et al. 2025).

The first global assessment on invasive alien species and their control by the Intergovernmental Science-Policy Platform on Biodiversity and Ecosystem Services (IPBES 2023) recommended an integrated governance approach to biological invasions that takes account of the above-mentioned factors. All the continental state members of the Southern African Development Community are members of IPBES. As members of IPBES, all these countries therefore formally approved the findings of the IPBES Invasive Alien Species Assessment (and have gone further, e.g. see the recently held Trialogue on Invasive Species Management between some of the Southern African countries, BESNet 2025). However, for an integrated approach to managing invasive ornamental plants in Southern Africa, it is important to consider the differences between the countries. Firstly, the extent to which each of the countries currently addresses biological invasions varies (cf. reports to the Convention on Biological Diversity, CBD Secretariat 2022), with South Africa standing out as a global hotspot in research and management (IPBES 2023). Secondly, an integrated governance approach across Southern African countries would need to reflect and consider the impact of national and regional interventions on the stakeholders involved since their collective engagement determines the success of management strategies (Shackleton et al. 2019b). Thirdly, matters of social-environmental ethics and justice would need to permeate the integrated governance approach to avoid entrenching and/or reproducing the colonial and apartheid legacies that persist (Ramutsindela et al. 2016).

Building on these premises, our goal in this paper is to explore what an integrated governance approach to biological invasions implies for Southern Africa’s ornamental horticulture sector. By reflecting on the actions and recommendations that key stakeholders shared with us, we aim to provide practical recommendations for more comprehensive management of biological invasions linked to the ornamental horticulture sector in Southern Africa. We link these recommendations to six strategic action areas proposed by the IPBES’ governance framework (IPBES 2023). These six actions of focus are:

- Resource innovation, research, and technology;
- Support information systems, infrastructures, and data sharing;
- Engage broadly across all stakeholders and Indigenous Peoples and local communities;
- Share efforts and commitments, and understand specific roles of actors;
- Enhance coordination and collaboration across international and regional mechanisms; and
- Improve policy coherence.

These six actions will be crucial components of the seventh action proposed in the IPBES report—the development of strategies at national and regional levels.

## Obtaining the views of the stakeholders involved

### The area of study

Our area of focus is Southern Africa as per the Southern African Development Community’s (SADC) definition. We limited our scope to the continental SADC members because they represent a geopolitical unit of 12 continental territories sharing tight historical, economic, socio-cultural, migratory, environmental, and political relations (Angola, Democratic Republic of Congo, Botswana, Eswatini, Lesotho, Malawi, Mozambique, Namibia, South Africa, Tanzania, Zambia, and Zimbabwe). These countries have commonalities in geographical-physical features (e.g. rivers, mountains), economy (e.g. trade routes, policies), politics (e.g. political boundaries, political systems), and culture (e.g. languages, religion). Our approach to Southern Africa serves as the right fit-for-purpose concept of a geopolitical region that accounts for the transboundary nature of plant invasions and ornamental-related trade and exchange (Rodríguez-Cala et al. 2025) while balancing our research capacity to cover a vast area.

### The methodological approach

This research followed an experiential, qualitative, and critical realist approach, where we see language as a reflection of people’s experiences (Bhaskar 1975; Braun and Clarke 2022). We find the notions of layered domains of reality of critical realism helpful in approaching the ‘humanness’ of biological invasions. Drawing from critical realist reflections (Price and Lotz-Sistka 2015; Price 2019), we contend that: (1) mechanisms can exist in a domain that is hard to access from the empirical alone; (2) the experienced realities are socially constructed and, hence, subjective and ever-changing; and (3) our research practice is also socially constructed and ever-changing, given that it is an intellectual and socioeconomic activity and livelihood subjected to power relations, cultural perceptions, social interactions, and political consequences (Toomey et al. 2017).

We used patchwork ethnography as the methodological umbrella (Günel et al. 2020; Günel and Watanabe 2024). This approach enables researchers to ‘patch’ together seams of data (or the moments where you generate them) when complete immersion and dedication to the fieldwork are unrealistic. Patchwork ethnography considers the researcher’s circumstances, making the research process more ethical and resilient. It especially accounts for the neoliberal conditions, environmental concerns, and women, queer, and neo-colonial contexts in which academic research takes place (Günel and Watanabe 2024).

### Data generation and interpretation

We generated data through: (1) online and face-to-face interactions with 135 people across seven countries (Botswana, Democratic Republic of Congo, Eswatini, Namibia, South Africa, Zambia, and Zimbabwe); (2) visits to 59 ornamental related sites (e.g. nurseries, landscaping businesses) in Botswana, Namibia, and Zimbabwe; and (3) participation in five events in Botswana, Namibia, South Africa and Zimbabwe (Table 1). 78 of the participants are environmental specialists, 30 are ornamental-related industry staff, and 24 are plant enthusiasts. The views expressed by participants on invasive species management more broadly and the trade, use, and management of ornamental species more specifically were interpreted in light of the IPBES’s six actions for an integrated governance approach to biological invasions.

**Table 1:** Participants per country and method of interaction to explore actions related to managing biological invasions from the ornamental sector in Southern Africa. Activities were conducted between August 2021 and May 2023

Country	Semi-structured interviews	Informal conversations	Observations
Botswana	Two environmental specialists	52 environmental specialists; 15 ornamental-related industry staff; 1 plant enthusiast	Annual Meeting of the Forestry and Range Resources Departments from Botswana [March 2023]; Visits to 29 sites
Democratic Republic of the Congo	None	Two environmental specialists	None
Eswatini	None	One environmental specialist	None
Namibia	Two environmental specialists	Two environmental specialists; Seven ornamental-related industry staff; 23 plant enthusiasts	Illustrated Talk session at the Botanical Society of Namibia [April 2023]; Visits to 10 sites
South Africa	Three environmental specialists	10 environmental specialists	Centre for Invasion Biology Meeting [April 2022]; South Africa National Symposium on Biological Invasions [July 2022]; No site visits
Zambia	Three environmental specialists	None	None
Zimbabwe	Two environmental specialists	Two environmental specialists; Eight ornamental-related industry staff	Annual Garden Show September 2022; Visit to 20 sites

Using purposive sampling (Robinson 2014), we reviewed academic publications and conference proceedings. We conducted scoping discussions with existing contacts to identify and recruit researchers, academics, scientists, university students, government officials, managers, educators, and activists working on a wide range of environmental topics, including biological invasions, from the mainland countries of the Southern African Development Community (SADC). We complemented that with a snowballing technique (Sullivan and Forrester 2019) to build on existing networks of research participants. These participants (referred to as 'environmental specialists' hereafter) have professional roles that historically position them as key knowledge holders (Turnhout 2024). They conduct research, inform and make management decisions, educate, raise awareness, and inform and enforce policies.

We also approached people whose income is linked to the trade and distribution of ornamental plants (referred to as 'ornamental-related industry staff' hereafter). Ornamental-related industry staff included those working for landscaping businesses, plant nurseries, and government offices managing urban spaces. This group, therefore, includes representatives from the private (including informal and formal businesses), non-profit, and government sectors. Initially, we located potential participants from this group (e.g. nurseries, landscaping businesses, garden centres) through Google Maps, and social media groups (e.g. Gabs Gardener [n.d.] in Botswana, Zimbabwe Gardening and Plant Lovers [n.d.], Flower Gardening in Malawi and around Malawi [n.d.], Gardening in and around Zambia [n.d.]). Later, we conducted a snowballing approach across the network of environmental specialists and industry participants. With this group, the focus was on Botswana, Namibia, and Zimbabwe, since these were the countries where we had the required network that allowed us to access the relevant stakeholder groups.

We included a third group (referred to as 'plant enthusiasts' hereafter) from the same three countries. We defined this group as those practicing ornamental gardening and/or interested in plants who are neither environmental specialists nor ornamental-related industry staff. For this group, we used a similar recruitment and snowballing approach for the ornamental-related industry staff group. Occasionally, participants from other stakeholder groups facilitated the recruitment of plant enthusiasts. These groups were defined as non-mutually exclusive, meaning that a participant could be part of more than one group. However, we assigned them to a specific group depending on the context in which we recruited them.

We interacted with environmental specialists, ornamental-related industry staff, and plant enthusiasts differently (details in Online Resource 1). Thirteen semi-structured online interviews were conducted with environmental specialists from Botswana, Namibia, South Africa, Zambia, and Zimbabwe through Zoom and/or WhatsApp. We visited ornamental-related businesses and institutions in Botswana, Namibia, and Zimbabwe; conducted semi-structured interviews and informal conversations; and made observations. We also carried out informal conversations and observations at the following events: Centre for Invasion Biology’s meeting (Stellenbosch, South Africa, April 2022), South Africa National Symposium on Biological Invasions (University of Fort Hare, Alice, South Africa, July 2022), Annual Garden Show (Harare, Zimbabwe in September 2022), the Annual Meeting of the Forestry and Range Resources Departments from Botswana (Gaborone, Botswana, March 2023), and an Illustrated Talk session at the Botanical Society of Namibia (Windhoek, Namibia, April 2023). In addition, we had informal meetings with staff members of the Department of Water Affairs, National Plant Health Unit, National Botanical Gardens, and Landscaping Departments of three city councils in Botswana. Other informal conversations occurred via email with environmental specialists, industry staff, and plant enthusiasts from the Democratic Republic of the Congo, Eswatini, Namibia, Zambia, and Zimbabwe. Relevant documents that participants mentioned (e.g. websites, social media, legislation, reports and academic publications) were tracked and consulted. We based each interaction on respect and written and/or verbal consent. However, the nature of some of the informal interactions did not always allow for formal consent as per our academic institutions’ guidelines, which can be impractical and inadequate (Maunganidze and Ruggunan 2021). Our fieldwork approach was flexible, guaranteeing feasibility and allowing for the best balance between reach and in-depth understanding while being culturally sensitive and appropriate (Macdonald 2024). Information for which we could not get formal consent, was therefore considered as an ethnographic experience for the main researcher.

The following central questions informed the above-mentioned interactions:

- What kind of actions aimed at managing biological invasions from the ornamental horticulture sector have you been involved in or are you aware of? How have they been implemented?
- What are your views about these actions, and how should they be implemented?

We recorded online interviews, face-to-face interviews, and informal conversations; otherwise, we took notes. Data were interpreted through thematic analysis. Our approach to this analysis is deductive since it was informed by the six actions (overarching themes in this case) in the IPBES’s framework (IPBES 2023). We explicitly looked for coding categories that fit these six overarching themes. Following our experiential qualitative and critical realist approach, we coded at a semantic level, meaning that we took what participants said as a reflection of their realities (Braun and Clarke 2022).

### Suggestions on how to approach this research

As previously stated, this research was based on critical realistic notions, following a qualitative approach. Unlike a quantitative positivist research, our approach is about interpreting human perceptions and opinions, which are socially constructed facts of the empirical domain of reality, as per the critical realist framework we used. We interpreted participants’ opinions and their reflections on strategies for the governance of biological invasions. The sample size was constrained by the nature of the research, its context, and the positionality of the main researcher (Rodríguez-Cala 2024). We are concerned with providing an enriching interpretation of the perspectives we had access to. In consequence, we do not claim to have provided a quantitative representation of each of the countries in the sub-region in this study, noting that approaching it from positivist and quantitative stances can raise problems of representation, generalisability, subjectivity, and bias (Braun and Clarke 2023a, b; Varpio et al. 2017, 2021).

## Views of stakeholders about the six IPBES recommendations for strategic action

In this section, we briefly unpack how the information shared by our participants aligns with the six strategic actions for the integrated governance of biological invasions (IPBES, 2023) and, thus, what the challenges are for implementing these actions. The participant names are changed throughout to maintain anonymity.

### Resource innovation, research, and technology

Academic and non-governmental institutions in Botswana, as well as government agencies from Botswana, Namibia, Zambia, and Zimbabwe, have worked on the documentation of the introduction history, distribution, impacts and the management of alien plants across all these countries (Table 2). However, it was clear that alien ornamental plants and alien plants, in general, are not a primary research focus. For example, an environmental specialist from Namibia explains:

**Table 2:** Examples of work documenting the introduction history, distribution, impacts and managing alien plants in Southern Africa

Focus country	Institution	Examples of outputs
Botswana	Okavango Research Institute Botswana University of Agriculture and Natural Resources National Botanical Garden of Botswana	Kashe et al. (2020) Mafokate et al. (2013)
Namibia	Namibia Botanical Research Institute Namibia Nature Foundation University of Namibia Namibian University of Science and Technology Botanical Society of Namibia	Chase and Pagad (2020) Iipinge (2019) Mutota (2018) Ndelitunga (2022) Njunge et al. (2017)
Zambia	Copperbelt University	Siachoono et al. (2021)
Zimbabwe	University of Fort Hare (South Africa)	Maroyi (2012) Maroyi (2017)

*[…] I think there needs to be a push to find out the extent of some of the distributions of some of these [alien] species. For instance, at the national herbarium, when we used to go out and in the past as well, people very rarely collected alien species because we know they are alien, and we do not generally house them, and with ornamentals as well. We do not go around collecting an ornamental because we know it is an ornamental […]* (environmental specialist Katy)

Several participants from environmental specialists and industry groups stated that additional research or ‘know-how’ on domestication/cultivation would be necessary to offer native plants as alternatives to the current alien ornamental plants in the market. Specialists from Zambia, the Democratic Republic of Congo, and Eswatini said there are not as many invasive species associated with the ornamental sector compared to the agricultural, livestock, and forestry sectors. By contrast, South African interviewees based in the Garden Route (Eastern Cape Province) and Kruger National Parks (Limpopo and Mpumalanga Provinces) emphasised the extensive research on the spread of alien plants used as ornamentals.

### Support information systems, infrastructures and data sharing

Data sharing through public platforms, like iNaturalist, is common in South Africa, where there is particularly rich data on ornamental alien plants. Researchers from the Centre for Invasion Biology at Stellenbosch University and other South African institutions are increasingly using records from iNaturalist to document and monitor the spread of alien species (e.g. Potgieter et al. 2024; Richardson and Potgieter 2024). A few iNaturalist users in Botswana actively contribute to documenting invasive plants, specifically garden escapes (@botswanabugs (2022)); we did not find examples of academic institutions using this platform for such functions to date. In that context, an avid user of iNaturalist and creator of the iNaturalist project on Garden Escapes of Botswana mentioned how useful this platform could be for academic institutions in Botswana for their research and outreach activities.

The Namibian Chamber of Environment (NCE), a membership-based organisation acting as ‘an umbrella association that provides a forum and mouthpiece for the broader environment sector, that can lobby with government and other parties’ (NCE n.d.), funds and supports a citizen-science project that focuses on ‘recording biodiversity and cultural heritage in Namibia’ (Atlasing in Namibia n.d.). This project has a specific section for alien plants and connections to the work that Cactus Clean-up group (Cactus Clean-up n.d.) carries out in Windhoek (Atlasing in Namibia n.d.). An undergraduate project at the Namibian University of Science and Technology produced an identification guide of the 50 most important alien plants in Namibia based on this tool from NCE (Mutota 2018). The organisation generally supports student projects (e.g. Iipinge 2019).

Social media gardening groups are also helpful platforms for data sharing across Botswana (Gabs Gardener n.d.) and Zimbabwe (Zimbabwe Gardening and Plant Lovers, n.d.). However, they are not widely used for topics like biological invasions, but more for providing gardening tips, learning from each other in making businesses and gardens, and advertising plant offers by businesses.

### Engage broadly across all stakeholders and Indigenous Peoples and local communities

Environmental specialists and people working in the sector, and, to a lesser extent, plant enthusiasts, see engagement across stakeholders and communities as a priority. They referred to engagement by mentioning education, awareness raising, knowledge sharing and persuasion. For example, the Botanical Society of Namibia has a consistent programme of awareness and education around invasive plants, which, amongst other things, organises educational activities, has an awareness campaign, and supports a citizen initiative that is currently managing cacti invasion across Windhoek, the capital of Namibia (Botanical Society of Namibia n.d.). However, the society is mainly comprised of ‘retired whites’ (environmental specialist Judith, Namibia); hence, it does not represent the wider Namibian society. Therefore, the society committee encourages ‘young blacks’ (environmental specialist Judith).

In Zimbabwe, every September, the Annual Garden Show in Harare focuses on using native plants, although each year has different themes. The co-organiser described how they have steadily persuaded businesses not to stock, use, or promote invasive plants in their business activities. Although, according to this interviewee, ‘our first 3 years, there was massive resistance from our exhibitors’ (environmental specialist Leyla), several businesses have followed and persuaded their customers to use native species alternatives to the usual alien ornamental plants. Furthermore, communication tools like the Zimbabwean Gardener Magazine have addressed the topic. However, most of the public attending the Annual Garden Show in September 2022 were white. In general, from across the region, participants suggested that wealthier and urban strata are more educated about the topic, being more mindful about what plants they use for gardening.

In Botswana, some businesses are conducting activities to address invasions (e.g. focusing on native plant species for gardening), especially those contributing to the SC Gardener Magazine. Botswana’s Department of Water Affairs has a consistent strategy where communication tools like TV, radio, and Facebook for the public and flyers and posters for government departments (e.g. landscaping units of the city council) are being used to raise awareness and tackle the spread of aquatic herbs (e.g. *Pistia stratiotes*, *Pontederia crassipes*, *Salvinia molesta*).

Regarding the formal education sector, a Zambian specialist said biological invasions are ‘a subject that is becoming popular in our curricula’ (environmental specialist Michelo). Namibian and South African specialists said that the level of inclusion of this topic in formal education activities relates to the availability of resources and capacity that schools have, which translates into perceived differences between schools, dependent on their affluence status:

*[…] from an educational point of view, I know it is part of the curriculum to learn about indigenous trees, which is quite good. But I think it’s also very dependent on the school. Some of the schools, which come from more affluent areas, they will then go to the National Botanical Research Institute, they’ll go to the botanical gardens, and they’ll be shown around. But the students that come from the less affluent areas don’t have that sort of exposure. I am not 100% sure of what is taught, but I guess it also depends on the resources available for the government schools. The level of education is fine in Namibia in some respects, but they don’t have the resources for a lot of things, and I do think it is what you are exposed to. […] When you’re more from the rural areas, it’s more about living in the moment and surviving and not always the sustainability […]* (environmental specialist Katy).

Participants often mentioned the need for better and consistent environmental education action plans at national levels, where communication strategies to reach people are improved sensibly. Referring to how messages come across to the gardening community in Zimbabwe, a participant who edits a gardening magazine reflected on the need for changing the discourse from belittling to empowering people with options, having concise and compelling messages, and being consistent in the long term:

*“I think we don’t talk about alien invasive plants in a manner that people are receptive to. Because we just heard naughty, naughty, naughty instead of going: “Hey, I see you’ve planted this. That’s actually really not very good for our indigenous environment. It does this, this, and this, but these are the other options that you can plant that will look just as good. And they have all these extra benefits: less water, less this, grow faster, this and this. Let’s say we just talk [making a gesture of scolding]. And people go: “I’m not listening to you”. […] And I think it’s going to be years and years of putting this information out there continuously. […] People are receptive to softer information in smaller bits. Then there is this massive information overload. I’ve noticed it as well in the magazine […] Because people are otherwise being so bombarded with information that they stop reading […]* (ornamental related industry staff Caroline)

Some participants noted that academics, scientists, and specialists are not usually trained to communicate complex issues to lay audiences. In that sense, the editor of the gardening magazine shared a related experience with staff from the agriculture department in Zimbabwe:

*[…] the agricultural people, for the nursery thing, they gave me a whole book a couple of months ago on invasive plants and nematodes. It is this [making a gesture with the hand] thick […] And they were like: ‘Oh, you should read it’. And I was like: ‘I’m really not gonna read that […], so you’re wasting your time giving me this info’. I wanted this, this, Bomp on one piece of paper! These are the top 10 invasive plants in Zimbabwe. Bomp!, bomp!…pictures, so we know what they look like. These are the best things you can plant in their places. Puff, puff, puff, pictures because people do not know what plants’ names are […]* (ornamental related industry staff Caroline)

### Share efforts and commitments, and understand specific roles of actors

During interactions with participants, it was clear how existing partnerships enable sharing efforts and commitments. For example, the Botanical Society of Namibia has a strong and consistent relationship with a nursery specialising in native species. The society is a member of the Namibian Invasive Alien Species Working Group, a recently created partnership between government and non-governmental bodies to reach national biodiversity targets that complement international commitments (NCE 2022). In Botswana, the National Botanic Garden, in conjunction with the Ministry of Environment, Natural Resources, Conservation and Tourism and academic institutions, has been running yearly workshops with nurseries to support each other and to promote knowledge sharing in plant growth, maintenance and the use of native plants. There is a solid network of businesses across Zimbabwe —and to a lesser extent Botswana— committed to promoting native species and to avoid the sale of invasive species (Box 1). Across such networks and groups, some degree of social accounting and reputation is linked to who sells or uses invasive plants and who does not. One of the Zambian specialists is working to create a similar network there.

One of the most palpable conflicts is between the formal and the informal businesses selling/using ornamental plants. Some formal businesses across Botswana, Namibia, and Zimbabwe perceive informal sellers as an unfair competitor because they do not pay taxes, licences, water bills, or rent and offer plants for cheaper prices. For a landscaper in Botswana, the problem is not ‘the little guys with little plants’ (ornamental related industry staff Petrus)—which his business supports—but the ones that ‘just come in, bringing a truckload of plants, drop it into the road and sell there, and there is no taxes’ (ornamental related industry staff Petrus). A small-scale backyard nursery owner in Botswana explained that people sometimes abuse the perceived vulnerability of road sellers by bargaining for lower prices. However, they would not do that to a well-established business or a franchise. Moreover, a backyard nursery owner from Botswana explained that the process for getting licences is bureaucratic and unclear, limiting the capacities of small-scale nurseries to be legalised and to tender for government and city council landscaping projects, on which several participants would have been keen. Several backyard nursery owners in Botswana were open for training and knowledge sharing. One nursery owner in Zimbabwe explained that informal sellers stocking invasive plants undermines her nursery’s efforts to educate their clients:

*[…] I think it’s the smaller roadside nurseries where they [clients] say: ‘Oh, but I saw it over that nursery. ‘And it’s like, ‘Yes, but they should not have it’. So, you are sometimes fighting a losing battle, but we have tried our best to eradicate all of that […]* (ornamental related industry staff Susan)

We observed that in Zimbabwe, for example, the conflict ‘becomes a racial thing sometimes’ (ornamental related industry staff Gerrit) since the road sellers are mostly ‘indigenous guys,’ whereas, as one of the interviewees and organisers of the garden show in Harare said, ‘a lot of our [formal] nurseries are white-owned’ (environmental specialist Leyla).

A nursery owner in Zimbabwe highlighted another type of conflict. According to this participant, a South African landscaper the nursery is working with on a large-scale project recommended plants that could not be legally used in South Africa under their Alien and Invasive Species Regulations (see Wilson and Kumschick [2024] for a review of the South African regulations). The participant criticised that people could go to Zimbabwe to do things they would not be allowed to do by law in South Africa, given the lack of legislation, law enforcement, and a higher level of corruption in Zimbabwe compared to South Africa. Other participants in Zimbabwe echoed the lack of legislation as a drawback. However, a nursery manager mentioned that legislation prohibited certain species (e.g. *Pontederia crassipes* [water hyacinth], *Jacaranda mimosifolia* [jacaranda], *Lantana camara* [lantana]) from being sold in Zimbabwe.

Another visible conflict is between businesses and government offices. Some businesses across Botswana and Zimbabwe complained about the government’s inability to enforce environmental laws and consistent programmes and the government staff’s poor training. One South African nursery owner based in Botswana found that government landscaping projects often ask for alien plants. When we asked relevant Batswana council officials about this, they responded that they must ensure plants are water-wise, fast-growing, unpalatable for free-roaming cattle, and appealing. Their options are limited.

According to a Zimbabwean specialist, government officers in Zimbabwe and other Southern African countries are not as skilled in the topic as in South Africa:

*[…] I need to stress how different that country [South Africa] is in its operation from the rest of Africa. And where it’s different is the government agencies actually function there. For example, when you speak to the environmental agency in South Africa, they will have people who are qualified, experienced, top of the field involved. When you talk to our Environmental Management Agency in Zimbabwe, you are talking to individuals who are kind of bottom of the professional pit. Because if somebody has a good qualification experience, or ability, they are not in government service […]* (environmental specialist Leyla)

Nurseries in Namibia and Zimbabwe complained about the legislative and bureaucratic obstacles to sourcing and selling native species, while it is easy to source and sell alien plants. In Botswana, several nursery owners also said that importing plants from South Africa is usually straightforward. A few nursery owners further explained that importing plants is usually more cost-effective for many small-scale businesses than growing many plants by themselves, given the high investment in infrastructure and resources that such projects require.

In contrast, in South Africa, several local professionals said that some nurseries sell plants banned by legislation, hindering the effectiveness of environmental agencies’ management actions and educational campaigns:

*[…] They [nurseries] are selling alien plants that they are not even supposed to be selling […] this also causes implications when it comes to management. Because it just makes things so difficult for the implementing managers to be on top of this problem. If you gonna get people promoting and selling them, on the other hand, you are busy trying to raise awareness: ‘Ok, no, not this way!’ And then, there are other people, on the other hand, selling them. So, it just makes things so complicated! […]* (environmental specialist Nofoto, South Africa)

There are also disconnections and controversies across the different sectors and offices of government that deal with plant and natural resource use, conservation, and management. Namibian and Batswana specialists mentioned the historical role that governmental forestry departments have had in promoting the use of alien plants. For instance, the landscaping units of a few city councils in Botswana described how they now have to manage plants that once were promoted by the forestry department. Nowadays, the forestry departments are shifting towards native plants in both countries. Forestry nursery branches in Tlokweng (Botswana) and Okahandja (Namibia) exemplify this. When interacting with several government departments in Botswana, it was evident that communication processes between them are not as effective as would be necessary for integrated governance of biological invasions.

Additionally, some department participants said they needed more support in training and funding. A common theme was that funding, and the state of countries’ economies limit the prioritisation of invasion management by stakeholders, especially governments. For instance, a Zambian specialist recalled how government-funded management has changed over the years since the independence, influenced by economic hardship, despite the large economical loss and impact caused by invasions:

*[…] Many years ago, after independence, there used to be greater pushes by the government to tackle certain noxious weed species like Lantana camara. And that, over the years, has gone by the wayside. At independence, they were quite good at still tackling those issues of invasive species. There were more agricultural extension officers that worked about going into the rural areas and helping in terms of best practices for cultivation, for soil conservation, as well as plant health and so forth. When Zambia hit economic hard times, those were things that disappeared, and they have never really returned […]* (environmental specialist Ryan)

Box 1 Nurseries committed to promote native plants in Botswana, Namibia and Zimbabwe. Photos by Diana Rodríguez-Cala
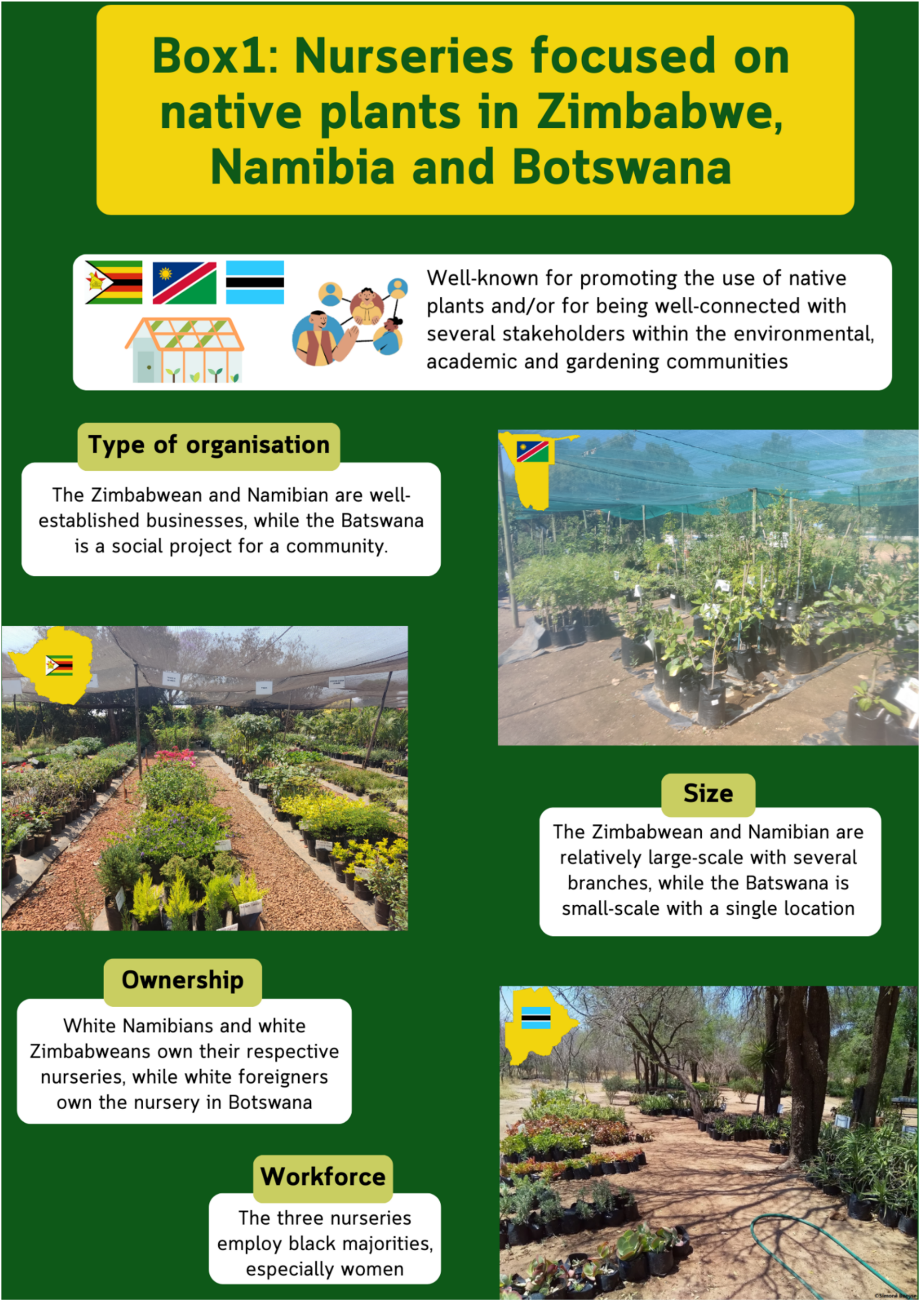

### Enhance coordination and collaboration across international and regional mechanisms

Environmental specialists recurrently mentioned regional collaboration as a priority. According to one South African specialist, regional collaboration across countries exists, but it has to move beyond the academic world toward the implementation of clear management actions:

*[…] I think they [regional collaborations] are happening. But they are more happening [at] collaborative research level. They do not go down deeper into the implementation stage. There is the collaboration when it comes to scientific writing, and so on, the meetings and stuff; they are there! But then, when it comes to the actual implementation, now it does not get there! So, it’s very important to start with them. It’s fine the collaborations, write the papers, the science and so on, that is fine, up until the end, up until like the implementation stage. So, it would be great if those collaborations actually could go down through the implementation stage […]* (environmental specialist Nofoto)

Another South African specialist could not recall examples of regional collaboration in preventing and controlling plant invasions linked explicitly to the ornamental sector. Based on this specialist’s interpretation, the lack of examples relates to the fact that research on the topic is still in the early stages, not enabling translation into management actions:

*[…] For ornamentals, I am not aware of, on the research side, many programmes or many projects even that are looking at this. It might just be my ignorance, but with the groups that I have worked with, or they do not know of, or I do not know of a huge amount of work on this topic, not nearly compared to what has been done on the other aspects of invasion. This is also then [related to] not being translated over into many management situations. A lot of it is still early stage, a lot of theories […]* (environmental specialist Owen)

Our interactions and observations across Botswana, Namibia, South Africa, and Zimbabwe suggest that collaborations amongst the countries are less intense than collaborations between each country and countries like the United States, Britain, Germany, and Australia. However, participants recalled collaborations in managing aquatic plants and cacti introduced as ornamentals between Botswana, Namibia, and South Africa. For instance, a Namibian specialist recalled when she participated in the joint (Namibian-Batswana) management of *Salvinia molesta* in the Okavango Delta region. Moreover, the Botanical Society of Namibia hosted a South African specialist in biocontrol to tackle the spread of several species of cacti, supported by the Cactus Clean-up group, a citizen-based control and monitoring programme (Cactus Clean up n.d.—mentioned earlier). This collaboration also involved a student project at the Namibian University of Science and Technology that assessed the effectiveness of those interventions (Ndelitunga 2022).

Specialists highlighted the need for increased regional collaboration in border inspection and control, legislation enforcement, knowledge sharing and training. Non-South African specialists emphasised the need to collaborate with South Africa regarding market adoption of native plants, public and sector outreach, training, and legislation design and enforcement. The shared perception amongst the non-South African participants is that South Africa has done these things better. Usually, the non-South African participants mentioned and considered South Africa as the provider of expertise. According to them, South Africa has been able to put its native plants into the market and take pride in its native flora for ornamental gardening and regulating plant sales like no other country in the sub-region. Likewise, this perceived superiority of South Africa was widely shared by the people working within the ornamental sector in Botswana, Namibia, and Zimbabwe (see Rodríguez-Cala et al. 2025 for more details). It was common to hear from businesses and institutions that knowledge about plant invasion management and/or invasive plants came from training and education in South Africa.

A Zimbabwean specialist mentioned essential challenges around the governance of regional collaborations when countries are in different developmental stages. Apart from the decisive influence of funding to sustain collaborations, this participant reflected on other factors. For him, the political-administrative infrastructure, the situation of Southern African countries, and the funders’ perceptions of these infrastructures and situations influence the capacity for collaborative management. In the context of transboundary conservation areas (Transfrontier Parks), which can be relevant for collaborative invasion management, the specialist explained:

*[…] [Greater Limpopo Transfrontier Park Contribution area] is not as useful as people would have liked it to be because it’s a platform where people just talk, but then, for things to be done, I think it all takes money. There is Big Brother South Africa, which stocks more money than Zimbabwe and Mozambique. If you hear about the stories in Kruger, the South African section, their roads are better, water is good, communication is better. Then, if you come to Zimbabwe or Mozambique, it is different. I think the platform is opening people’s eyes and sharing ideas of what and how things can be done, but then, for things to really happen, I think it takes more than just that. […] South Africa has a better economy, so their operations are well funded by the national fiscus. Their national budget is better than Zimbabwe’s. Because things work [in South Africa], it attracts more professionals to stay in the system and all that. […] I think Zimbabwe, in first value, would attract more funding because of the economy and poverty, but then there are issues that come into play, the politics and all that, the accountability, that makes people not bringing the funding […]* (environmental specialist Tinotenda)

Furthermore, this specialist added that having an alliance with an ‘international organisation’ has proved to be a fit-to-purpose strategy to make the attraction of international funding smoother:

*[…] we are not government [only], but we are a national park in partnership between an international organisation and the government. It makes people know that if I put my money into this organisation, I am not giving to the Zimbabwean government, but I am giving to an organisation that is being managed by—from some angle—an international organisation, which is world-renowned, with a good track record of use of funds and all that […]* (environmental specialist Tinotenda)

### Improve policy coherence

Concerning policy, most non-South African specialists and some businesses have expressed that a specific legislative framework is needed. Most of them acknowledged that their countries’ biosecurity laws focused on the phytosanitary implications of plant imports, not specifically tackling biological invasions. Two specialists—from Namibia and Botswana—mentioned laws that, although not specific to biological invasions, include the management of functional groups of species like agricultural or aquatic weeds. Such legislation includes, for example, the Aquatic Weeds Act 1989 in Botswana and the Agricultural Pest Act 1973 in Namibia (see further examples in Online Resource 2 and a case study from Namibia referred to wetland management [Bethune and Ruppel 2007]). One participant from the National Plant Health Unit in Botswana said that a nursery bill to control the plants that nurseries can import had been drafted for approval at the moment of our interaction.

A Namibian specialist summarised related laws—some of them from the times of the colonial Apartheid South African regime—and policies in place:

*[…] Our constitution in Namibia can be interpreted as asking us to deal with the issue. We have an environmental clause in the Constitution, Article 95* *L […], [which] we can interpret as saying that the maintenance of the overall ecosystem is a national obligation, and depending on all actions of the legislature, and this includes the control of invasive alien species, where [they] pose a threat to an ecosystem or ecological process, or biological diversity or the welfare of people or to sustainable utilisation of natural resources. We also have more specific legislation in the Ministry of Environment and Tourism policy, so they are aware of the alien invasive issue, which is being thoroughly addressed in the new parks and wildlife management tool. There is also a Forestry Act, which looks at the introduction of alien invasive species, and that it was recently revised. The Ministry of the Department of Agriculture keeps a list of species not allowed into the country, where the five water weeds are specifically mentioned, and then cacti are just as a general concept […] The Environmental Management Bill provides some very good contingencies. It specifically states the application of a precautionary principle under the section on principles of environmental management […], and it is a legal requirement to do EIA [environmental impact assessment] before any development. […]* (environmental specialist Sandra)

This participant added that despite these many laws, there are problems in their coordinated enforcement across societal sectors (e.g. forestry, tourism, environment, agriculture) and in the design and implementation of complementary policies. Interestingly, the specialist mentioned the counteraction that the Forestry Act might have in the management of a specific invasive plant in Namibia, denoting incoherencies between closely related sectors and institutions:

*There is a clause in the Forestry Act that provides the protection of the environment and makes it an offence ‘to harm, injure, remove any living tree bush or shrub within 100 metres of any river stream or watercourse’, but that is actually something counter to what we need to do when removing riverside aliens such as Prosopis [Neltuma]. It essentially makes it illegal, and therefore, we need to have these excluded from that type of legislation. […]* (environmental specialist Sandra)

Moreover, as described earlier, in Zimbabwe, laws are not necessarily well-known amongst participants, even amongst environmental specialists. Overall, non-South African participants praised South Africa’s legislative corpus on alien species, deeming it the model to follow in the sub-region. However, the South African participants recognised both strengths and weaknesses of the South African legislative framework. One specialist said the South African legislation was too general and extensive, limiting its operationalisation, especially when building capacity amongst the government staff that is supposed to enforce it. This participant further suggested that the species listing and derived management categories could adopt a local approach that allows stakeholders to assess which species are included and what management measures to take, considering the local context. A different South African informant said that the high levels of corruption across the South African governmental administration do not enable effective enforcement. Furthermore, another South African specialist commented that the South African legislation had not been designed to serve and protect non-privileged South Africans, hindering the capacity to reach the economy’s informal sector effectively. In addition, some business owners complained about the lack of a long-term vision of policies and the general corruption and instability in Africa compared to ‘first-world’ countries.

### Recommendations

Building on six of the strategic action areas for an integrated governance approach to biological invasions proposed by IPBES (2023), this paper—to our knowledge—constitutes the first attempt that examines how this framework applies to a specific area and sector since the launch of the IPBES report. The process, although straightforward in general, turned complex when distinguishing overarching themes between ‘Resource innovation, research and technology’ and ‘Support information systems, infrastructures, and data sharing,’ and between ‘Engage broadly across all stakeholders and Indigenous People’ and ‘Share efforts and commitments; understand specific roles.’ These strategic actions (or overarching themes) overlap, which is expected, given the integrated nature of governance and the multi-factorial and cross-sectorial nature of biological invasions.

This study found significant efforts are ongoing in Southern Africa to address biological invasions from the ornamental sector. However, more integration and consideration of the broader geopolitical and socio-historical context would be beneficial (Table 3). If the existing limited funding for invasive species management were channelled to better coordination, such programmes could integrate all the actions and actors described in this paper while facilitating policies and practices to manage biological invasions.

**Table 3:** Summary remarks of what the strategic action areas for an integrated governance approach to biological invasions proposed by IPBES (2023) appear to be in Southern African countries

Strategic action area	Summary
a. Resource innovation, research, and technology	While research and innovation activities are taking place in several Southern African countries, the attention to biological invasions linked to the ornamental sector varies substantially between countries, giving room for more integration and focus.
b. Support information systems, infrastructures, and data sharing	Data and knowledge sharing are happening with differences in scale and type of platforms and infrastructures across countries. There is great potential for more topic integration into such platforms across Southern Africa.
c. Engage broadly across all stakeholders and Indigenous Peoples and local communities	Although this action (overarching theme) is viewed as a priority for addressing invasive plants and various activities ranging from education and awareness to persuasion and knowledge sharing are happening in Botswana, Namibia, and Zimbabwe, challenges remain in ensuring inclusive participation and effective communication across stakeholders.
d. Share efforts and commitments; and understand specific roles of actors	There are joint efforts and commitments between environmental organisations, governments, and businesses in several countries. However, conflicts arise between formal and informal plant businesses, and businesses and government agencies. Issues in regulations, bureaucratic hurdles, communication, and inequalities shape these conflicts. Furthermore, poor funding and resources, economic instability, as well as communication issues limit what government units can do.
e. Enhance coordination and collaboration across international and regional mechanisms	Regional collaboration, beyond academic research to management actions, is still scarce across Southern Africa. Challenges such as perceptions of superiority and inferiority, economic disparities, legislative inconsistencies, and the need for solid governance and funding hinder effective collaboration across the region.
f. Improve policy coherence	Policy coherence faces challenges across the sub-region, especially concerning cross-sectorial and societal coherence, enforcement, and inclusivity. South Africa’s legislative model on alien species is praised amongst non-South African specialists, although several chal;lenges are noted, particularly by South Africans, e.g., insufficient capacity building and inclusivity.

**Fig. 1:**
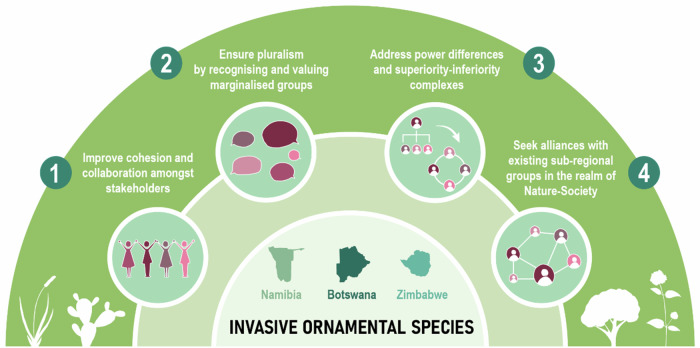
Recommendations for facilitating conflict management and decision-making in managing ornamental-related biological invasions across Southern Africa, focusing on the three countries where more interactions occurred in this research. Illustration by Verónica Barrajón-Santos

We unpacked these needs in four practical recommendations (Fig. 1) that we hope contribute to the ongoing efforts to tackle biological invasions in Southern Africa.

- Improve cohesion and collaboration amongst stakeholders by using the framework for stakeholder engagement proposed by Novoa et al. (2018). Starting with Botswana, Namibia, and Zimbabwe—given the above-presented deeper understanding of the contexts therein—regular communication spaces (for example, Living Labs) could be used where environmental specialists, ornamental-related industry staff, and plant enthusiasts meet to debate the management of biological invasions. Such spaces for dialogue could happen in the form of workshops, meetings, and by using already created spaces for instance, the Annual Garden Show in Harare, the Illustrated Talks by the Namibian Botanical Society, and forestry/environmental workshops like the one hosted by the National Botanic Garden and Forestry Departments in Botswana. However, such existing spaces might limit the reach of informal businesses, especially in Namibia and Zimbabwe—we recommend the creation of mindfully organised new spaces so that informal businesses can join and feel safe enough to share their knowledge. Such spaces should include a mix of stakeholders: government, private businesses, academia and non-profit organisations, etc.
- Ensure pluralism, where ‘non-scientific’ and subaltern views and practices from historically marginalised social groups in Southern Africa are recognised and valued as significant knowledge and realities (Bandauko and Arku 2024; Toomey et al. 2017; Turnhout 2024; Zein-Elabdin 2009). Designing suitable communication spaces requires carefully considering the segregation and marginalisation issues within the ornamental sector, their historical structural causes, and discussing the overall social contexts and embedded power dynamics we aimed to show. For this to materialise, there should be a paradigm shift in policy and governance that includes and considers the informal sector through inclusive and participatory urban planning (Bandauko and Arku 2024), and in legislation that changes the colonial laws still in place (Mitullah 2003; Wily 2011). Zein-Elabdin’s (2009) notions of profound economic, cultural, and institutional mixing (i.e. hybridity) into legislative and policy decision-making could benefit the movement towards fairer, more ethical, and more locally relevant legislations and policies. This incorporation means that the historical and geopolitical mechanisms that determine the inequalities and power dynamics in the sub-region should be recognised, even if this recognition produces discomfort.
- Address the power differences and superiority/inferiority complexes that the representatives from the different countries in the sub-region unconsciously might carry when taking an invasive management programme to a sub-regional level. For sub-regional cooperation to become more effective, collaborators need to pay attention to the nuances constructed along the history of the sub-region and the diversity of cultural and socio-geographical contexts. In particular, South African collaborators should be mindful of this power relation where South Africa acts as ‘the big brother’ and the giver of expertise to avoid the naïve and good-intentioned imposition of strategies that might be counterproductive and unfair for the other partners. Moreover, such processes should be careful not to disproportionately increase South Africa’s perceived superiority in the topic.
- Collaborate with existing sub-regional groups working in nature-society, like the Southern African Program on Ecosystem Change and Society (SAPECS n.d.) and the Environmental Education Association of Southern Africa (EESSA n.d.). Both groups are actively working towards more sustainable, just, and equitable futures in the sub-region of socio-ecological research and practice in Southern Africa (Biggs et al. 2022; see EESSA’s publications in EESSA n.d.). Unsurprisingly, South Africans and/or people based in South African institutions lead EESSA as the majority in SAPECS. This situation, again, is an artefact of the power dynamics in the sub-region—where South Africa plays a dominant role—that should be considered when framing collaborative networks. Creating a Southern African Symposium on Biological Invasions where stakeholders can share efforts, concerns and commitments, could be a space to network and frame joint efforts between countries.

## Conclusions

This study interprets what an integrated governance approach to biological invasions appears to be in Botswana, Namibia, and Zimbabwe (with some insights for the broader Southern African region), based on the strategic actions proposed by the IPBES report on invasive alien species and their control (IPBES 2023). Building from our interpretation, we provide practical recommendations to foster such an approach for the ornamental industry and ornamental-related plant invasions in Southern Africa. These recommendations could facilitate conflict management and decision-making, emphasising the need to consider the power imbalances reflected at the sub-region’s individual, group, societal, and regional levels. Moreover, our recommendations could also be applied to similar sectors like the pet industry, whose significance in animal invasions is still under scrutiny in South Africa (e.g. Martin and Coetzee 2011; Nelufule et al. 2020; Shivambu et al. 2020).

Our recommendations attempt to tackle the power imbalances and inequalities derived from socio-historical and geopolitical processes by proposing transparent and participatory engagement, building on stakeholders' the commonalities. Such recommendations need to be developed so that, on the one hand, they allow for regional cross-country coordination and, on the other hand, enable each country to develop and implement its strategy. Likewise, an appropriate legal framework that serves nature and people as part of nature must be co-designed, shared, and adequately implemented to facilitate actions.

Environmental specialists are likely to be the facilitators of the abovementioned endeavours. Thus, as environmental specialists, we should acknowledge that our perceptions, attitudes, preferences, behaviours, and public discourse have the potential to significantly influence other stakeholder groups because we have been historically positioned as key knowledge holders (Pascual et al. 2021; Toomey et al. 2017; Turnhout 2024). Despite its apparent rigour, scientific work is a livelihood subject to the same forces as any other (Bhaskar 1975; Pascual et al. 2021; Price 2015; Toomey et al. 2017) and the western thinking and lifestyle dominate it (Rogers et al. 2013; Rodríguez-Cala 2025).

Reiterating Pascual et al. (2021) and Turnhout’s (2024) calls, we argue that environmental specialists need to work towards improving how our institutions are governed and our environmental practice is conducted to counteract power concentration and marginalisation within the sector and society. We also need to embrace open-mindedness, practicing a relational approach to environmental practice and communication, where our knowledge and opinions do not become hegemonic over others. This process would be the first step toward the transformative change necessary for tackling the current environmental crisis’s underlying political and economic causes (IPBES 2024).

## Supplementary information

Online Resource 1

Online Resource 2

## Data Availability

No datasets were generated or analyzed during the current study.
