# Supplementary material for: Ornamental horticulture in Southern Africa: strategic actions to address biological invasions
Source: Environ Manage. 2025 Aug 28;75(12):3203–19. doi: 10.1007/s00267-025-02241-y (PMC12575546; doi:10.1007/s00267-025-02241-y)
Supplement: Supplementary file 1 — Online Resource 1 [file 267_2025_2241_MOESM1_ESM.pdf]

Online Resource 1

Title: Ornamental horticulture in Southern Africa: strategic actions to address biological invasions

Diana Rodríguez-Cala[1,2,3], Jana Fried[1], John R. U. Wilson[4,3], Katharina Dehnen-Schmutz[1], Seoleseng O. Tshwenyane[2], Israel Legwaila[2]

[1]Centre for Agroecology, Water and Resilience, Coventry University, Coventry, UK

[2]Department of Crop and Soil Science, Botswana University of Agriculture and Natural Resources, Gaborone, Botswana

[3]Centre for Invasion Biology, Department of Botany and Zoology, Stellenbosch University, Stellenbosch, South Africa

[4]South African National Biodiversity Institute, Kirstenbosch Research Centre, Cape Town, South Africa

Corresponding author's email: Diana Rodríguez Cala, dianarodriguezcala@gmail.com/ rodriguezd@uni.coventry.ac.uk

SI1: Recruitment and data collection

The approach to recruitment followed a purposive sampling strategy (Robinson 2014) that nested stakeholder groups into three geographical scales. Environmental specialists were recruited from mainland countries in the Southern African Development Community (SADC). SADC's definition corresponds to historical, economic, socio-cultural, migratory, and political relations amongst the 16 member countries, thus serving as the right fit-to-purpose concept of the geopolitical region that accounts for the transboundary nature of plant dispersal and ornamental-related trade. This definition allowed for the sub-regional perspective the research attempted to cover. The ornamental industry staff and gardeners were recruited from Botswana, Namibia, and Zimbabwe, the countries where the research zooms in deeper. Recruitment and data collection began online in August 2021, focusing on environmental specialists, when COVID-19-related travel restrictions were in place between the UK and Southern Africa. After restrictions eased, a second phase of recruitment and data collection of the three stakeholder groups occurred between May 2022 and August 2023 in South Africa, Botswana, Zimbabwe, and Namibia.

Data collection consisted of semi-structured and structured interviews (questionnaire-based), workshops, informal conversations, surveys (online and paper-based questionnaires), participant observations, and consultation of relevant websites and/or documentation from our

participants. The combination of methods was adapted depending on the geographical area and the type of stakeholder. Moreover, the data collection started when the COVID-19-related restrictions were still in place. Thus, we designed it to be as flexible and resilient as possible, considering both participants and their circumstances regarding relative geographical location, time availability, our capacity to access the participants' locations, internet access, security issues, and comfort.

Environmental specialists

For environmental specialists, semi-structured interviews, informal conversations, and two workshops were used (Table 1). Initially, potential participants were identified through a review of subject-relevant academic publications, project reports, online databases, and participant lists from relevant regional events. Exploratory online/email conversations with relevant professionals in Namibia, Botswana, and South Africa helped identify further contracts among their professional networks. From August 2021, potential participants were contacted through email to hold online semi-structured interviews with the project's rationale in a participant information sheet and an informed consent form to be signed off. Subsequently, more participants were recruited following a snowballing approach whereby an approached potential participant would be asked to name another potential participant even if they replied, refusing to hold the interview.

Table 1: Sample of environmental specialists by country, occupation and data collection method. In occupation, 'researcher' refers to researchers based in non-academic institutions like non-governmental organisations, protected areas, private businesses, etc.

ID	Country	Occupation	Data collection method
1	South Africa	manager, researcher, practitioner	online semi-structured interview
5	South Africa	manager, practitioner	online semi-structured interview
6	South Africa	academic researcher	workshop (face-to-face)
10	Zambia	academic researcher	online semi-structured interview
11	Zimbabwe	academic researcher	online informal conversation
12	Botswana	academic researcher	online semi-structured interview
13	Zambia	academic researcher	online semi-structured interview

15	South Africa	manager, researcher, practitioner	online semi-structured interview
17	Namibia	Researcher	online semi-structured interview
21	Namibia	academic researcher	online semi-structured interview
26	Zambia	manager, practitioner	online semi-structured interview
29	Zimbabwe	activist, practitioner	online semi-structured interview
30	Botswana	academic researcher	online semi-structured interview
32	Zimbabwe	manager, researcher, practitioner	online semi-structured interview
34	South Africa	academic researcher	workshop (face-to-face)
35	South Africa	academic researcher	workshop (face-to-face)
36	South Africa	academic researcher	workshop (face-to-face)
37	South Africa	postgraduate researcher	workshop (face-to-face)
38	South Africa	academic researcher	workshop (face-to-face)
39	South Africa	postgraduate researcher	workshop (face-to-face)
40	South Africa	postgraduate researcher	workshop (online)
41	South Africa	postgraduate researcher	workshop (online)
42	South Africa	postgraduate researcher	workshop (online)
43	Botswana	researcher	face-to-face informal conversation
44	Botswana	manager, practitioner	face-to-face informal conversation
45	Zimbabwe	researcher	informal conversation through email

46	Botswana	governmental officer, manager	face to face informal conversation
47	Botswana	manager	face to face informal conversation
48-52	Botswana	governmental officers, managers	face to face informal conversation through a meeting
53-94	Botswana	governmental officers, managers	open fora workshop

Of the 33 potential participants contacted through email who covered the continental members of SADC (Angola, Mozambique, Tanzania, Democratic Republic of the Congo, Zambia, Zimbabwe, Namibia, Botswana, Eswatini, Lesotho and South Africa), 12 interviewed five of those countries: Zambia, Zimbabwe, Namibia, Botswana, and South Africa. The other two from this initial recruitment process interacted with the primary author in a later workshop held in South Africa (ID 6) and online informal conversation (ID 11). Initially, participants with ID 43 onwards were not recruited for online interviews, but we interacted with them through snowballing while in Southern Africa (Table 1).

Semi-structured interviews

A guide for the semi-structured interviews was designed for up to one hour. It was calibrated with two pilot interviews held with two African environmental specialists before the start of data collection. Interviews flowed differently each time, and participants could focus on other related aspects of their experiences. Most were held on Zoom, ranging from 45 minutes to one hour. Two of them were held on WhatsApp, resembling a chat where voice notes and text were used at the participants' pace, meaning they could continue the chat whenever they had the chance. One had poor internet capacity to hold a Zoom meeting, whereas the other did not have time. Two of the interviewees from Botswana became research advisors and authors of this paper. One interviewee from Namibia and another from Zimbabwe became research collaborators.

Workshop

The workshop occurred at the Centre for Invasion Biology group meeting in May 2022. The leading researcher (DRC) gave the project's rationale in a PowerPoint presentation. We used a

version of the perception exercise “Past, present and future” (Salas et al. 2010) to identify the participants’ perceptions of the role of the ornamental horticulture sector in plant invasions in South Africa. The participants took part online and face-to-face (Table 1) and were asked to make notes of their responses and stick them to flip charts that I had already created with mind maps of codes from previous interviews. The flip charts were revealed after the discussion so participants could stick their notes.

Informal conversations

These included email exchanges and face-to-face and online conversations. For example, interactions with participants through email about the topic were taken as informal conversations. Face-to-face and online meetings that happened spontaneously were taken as informal conversations since there was no opportunity to structure them as interviews, but they contained relevant data.

Ornamental industry related staff and Plant enthusiasts

Questionnaires, informal conversations, and participant observations were used with ornamental industry staff. Initially, potential participants from the sector were explored on Google Maps and social media groups during the scoping phase of the project before data collection started officially. Later, some key informants, such as the leading researcher (DRC), held interviews and directed us to potential participants within the Botswana, Zimbabwe, and Namibia sectors. Regarding the ornamental gardeners, the time spent in Southern Africa allowed the leading researcher (DRC) to build relationships, find spaces to divulge the questionnaire to the public, and interact with people about the topic.

In June 2022, the leading researcher (DRC) arrived in Botswana after establishing an agreement with the Department of Soil and Crop Science from the Botswana University of Agriculture and Natural Resources. They signposted potential participants in Gaborone, Francistown, Selebi-Phikwe, and Maun. Additionally, the leading researcher lived in Botswana, and the recruitment combined snowballing among participants, friends, and neighbors, searching in street advertisements and classified advertisement magazines. One participant indicated the directory of the gardening magazine “SC Gardens Botswana.” Another permitted posting of online surveys in the Gabs Gardeners Facebook group for the sector and the public. WhatsApp was primarily used to contact participants. The circumstances allowed semi-structured interviews, questionnaires in online and paper form, informal conversations, and extensive observations and experiences with multiple stakeholders related

to the sector. The online survey for ornamental gardeners was advertised in WhatsApp groups and through friends' and participants' WhatsApp statuses and other social media. Facebook posts, stories, and Whatsapp statuses are common social platforms in Botswana across all ages. The one-week courses on Floriculture, Flower Arrangement, and Landscaping that the Botswana University of Agriculture and Natural Resources offers for the public were also used to advertise the online and paper-based questionnaire versions. Additionally, three researchers contributed to the SC Gardens Botswana Magazine with a short article in which the survey for ornamental gardeners was advertised.

In Zimbabwe, recruitment and data collection occur differently. One of the Zimbabwean specialists that the leading researcher (DRC) interviewed online co-organises the Annual Garden Show in Harare, to which she invited DRC in 2022. In September 2022, DRC spent 13 days in Harare, attending the garden show and experiencing the sector beyond the show. She conducted interviews, informal conversations, and extensive observations in and beyond the show around Highlands, Borrowdale, Chisipite, Greendale, Cranbone, and Mount Pleasant, visiting and interacting with people in ornamental-related establishments. All of those neighbourhoods are considered low-density wealthy suburbs. The leading researcher could not reach the high-density neighbourhoods because of safety reasons. The online questionnaire was distributed through online platforms like WhatsApp and the Zimbabwe Gardening and Plant Lovers Facebook group. In addition, DRC contributed to the Zimbabwean Gardener Magazine with a short article in which the online questionnaire for end users was also advertised.

Data collection in Namibia occurred in April-May 2023. A previously interviewed Namibian specialist put us in contact with the Department of Natural Resource Management at the Namibian University of Science and Technology (NUST) and the executive board of the Botanical Society of Namibia. DRC was invited to give a guest lecture to the Hons students of Natural Resources Management and a talk to members of the Botanical Society of Namibia within its "Illustrated Talks" program. Both opportunities were used to collect data from the Hons students' families and relatives with paper-based questionnaires. The talk for the Botanical Society of Namibia was made through focus groups. Key informants and collaborators from the Botanical Society of Namibia helped contact potential participants and visit establishments around Windhoek and Okahandja. Site visits were complemented with informal conversations and observations. Business directories were used to reach out to businesses through WhatsApp and email with the link to the online version of the questionnaire.

Questionnaire (online and paper forms)

Two types of questionnaires were designed for the industry staff and end users. The questionnaire for the sector was co-designed with one of the environmental specialists from Zimbabwe, who co-organises the Annual Garden Show in Harare. After consultation with other researchers and piloting, three versions of the two questionnaires for each of the three countries were made to account for the differences in administrative organisations and others. For example, the questionnaire for ornamental gardeners in Botswana specified that respondents could refer to the house they preferred to talk about if they did not have time to fill out the questionnaire twice because people residing in the capital usually have a house in the village. Given that more time was spent in Botswana, the questionnaire was translated into Setswana, the national and most spoken language. Additionally, respondents were invited to respond in Setswana if preferred. Unfortunately, the same could not be done for Namibia and Zimbabwe.

The questionnaires for the industry staff and end users had three sections. This paper focuses on the first two sections of both questionnaires. For the sector, the following aspects are central: businesses' structure and customer base, factors influencing the businesses' operation, bestseller plants, and supply chain. For the end users, we asked people to describe their spaces, what their favourite plants are and why, where they source their plants from, how they maintain their spaces with plants, and the criteria people use to choose plants for their spaces.

Informal conversations coupled with participant observations

These occurred when visiting ornamental-related establishments and during daily life in the three countries. Every possible interaction related to the topic was journalled. A record of WhatsApp and email exchanges on the topic was kept. Some of these interactions guided the data collection to websites and online media, which participants related to when talking about invasive plants (e.g., INaturalist, nurseries' websites, and initiatives' websites). Therefore, these conversations and observations diversify into different aspects of how the sector operates from the perspectives of ornamental industry staff and end users, the sector's dynamics, factors influencing the sector's operation, and people's preferences.

Focus group

Focus groups were held with 23 persons attending the Illustrated Talk "Exploring the ornamental gardening sector in Southern Africa with a critical environmental lens" (Botanical Society of Namibia, 2023) in April 2023. The audience was divided into three groups or teams

to answer six questions about their favourite plants, the places where they source such plants, and the challenges they faced when gardening. We finalised the session with collective recommendations to face those challenges.

References

Robinson OC (2014) Sampling in Interview-Based Qualitative Research: A Theoretical and Practical Guide. *Qualitative Research in Psychology*. https://doi.org/10.1080/14780887.2013.801543

Salas MA, Tillmann HJ, McKee N, Shahzadi N (2010) Visualisation in participatory programmes: how to facilitate and visualize participatory group processes. Southbound.

Botanical Society of Namibia (2023) Illustrated Talks: Diana Rodriguez Cala. Illustrated talk: Diana Rodriguez Cala | Botanical Society of Namibia. https://botsoc.org.na/whats-on/illustrated-talks/illustrated-talk-diana-rodriguez-cala

SI2: Semi-structured interview guide

Hello/Bon dia/Bonjour, very happy to see you at least on the screen! How do you feel today?

[Pay attention to the interviewee's response and make the interaction flows with body and verbal languages. Here particularly make them feel comfortable and in a informal conversation]

My name is Diana Rodríguez Cala, I am from Havana, Cuba (at the other side of the Atlantics ☺). As you might have already read from the Participation Sheet and Informed Consent Form, I am doing a PhD at the Centre for Agroecology, Water and Resilience from Coventry University in England, partnered with Stellenbosch University in South Africa. I would like to look at how the ornamental sector might be influencing plant invasions in Southern Africa. With this interview, I would like you to share with me your perceptions, in particular your risk perceptions, about invasions by alien ornamental plants in your territory and region. These interviews are just an initial stage of a broader project that seeks to understand how people perceive and use alien ornamental plants, their perceptions about the management of invasive species derived from the ornamental sector, as well as their risk perceptions about the capacity of these species to become invasive in Southern Africa. I am hoping that the results of this research are beneficial for the management of plant invasions in Southern Africa and for the collaboration across this extended network of professionals that I am getting to know now. It

is important to clarify that I am interested in your impressions and views both as professional but also as a human who has a life full of multiple experiences and perceptions.

To comply with the ethical code, I would need you to give your informed consent to participate in and video recording the interview once I start to record. I need to have your consent actually recorded. May I start recording now?

[Wait for the interviewee to confirm]

Before we formally start the interview, would you mind to verbally confirm your informed consent for participating in and video recording this interview of about 1 hour and a half, for the purpose of this project?

[Wait for the interviewee to confirm]

In addition, would you mind to confirm that you are happy for me to save your contact details for a potential further follow-up, sharing the results of all the phases of this project once they are published and collaborating in the future?

[Wait for the interviewee to confirm]

[Pay attention to the interviewee's response and make the interaction flows with body and verbal languages]

Now, just for me to have a first-hand glance of your experience in the environmental sector, can you tell me a little bit about your training background? Kind of your work story summarized.

[Pay attention to the interviewee's response and make the interaction flows with body and verbal languages. Here, particularly show excitement and interest about her/his story.

Make sure you listen to and pick up on the elements of which the interviewees are saying in the subsequent questions so that the interview is more a conversation where these questions come out from what they are expressing]

So…based on your experience of work and life, how can you describe the issue of plant invasions in your territory and region: how would rank it among other environmental issues in your context?

Now, I would like to discuss with you about the influence of the ornamental sector on spreading alien plants across your territory and region.

Can you comment on how do you think the ornamental sector could be influencing plant invasions in your territory and region?

How would you classify the role of ornamental trade/exchange in plant invasions, as pathway of introduction and/or pathway of spread?

What do you think are the mechanisms and dynamics by which the ornamental sector influence plant invasions in your territory and region?

How do you foresee the contribution of the ornamental sector to plant invasions in your country?

Further cue: Will it gain more importance or not? Why?

According to the African Union, a free trade zone is being created to promote trade and economic development across the continent. How do you think the potentially increasing trade between African countries will influence the trade/exchange of alien plant species, specially the ornamental species?

Now, I would like to know your thoughts of the general perception of the public your territory and region about alien plants, particularly the alien ornamental plants.

How do you think people in your territory and region perceive alien ornamental plants with respect to the native counterparts?

How do you think these perceptions vary across different human populations in your territory and region?

Further cue: differences in aliens use as ornamental between rural people vs. urban people, indigenous people vs. mixed-culture descendants, high-income people vs. low-income people.

To what extent do you think the public in your territory and region is aware that ornamental alien species have the potential to become invasive?

How do you think this awareness vary across different human populations in your territory and region?

Further cue: differences in risk awareness between rural people vs. urban people, indigenous people vs. mixed-culture descendants, high-income people vs. low-income people.

In your opinion, what features make a plant appealing as ornamental for people in your territory and region?

How do you think these preferences vary across different human populations in your territory and region?

Further cue: differences in plant preferences between rural people vs. urban people, indigenous people vs. mixed-culture descendants, high-income people vs. low-income people.

Finally, I would like you to share your opinions and experiences with me about the stage of the research, management, control and prevention of invasive plants in your territory and region, specifically the ones considered ornamental, as well as the actions you believe are necessary to tackle plant invasions, particularly from the ornamental sector, in a more effective way in your territory and region. These following questions will be the last few of this interview.

What is the legislative framework in your territory and region with respect to the prevention, management and control of invasive plants?

What do you think about the framework in terms of effectiveness, enforcement and compliance?

What do you think are the best approaches to tackle the introduction and spread of alien species from the ornamental sector in your territory and region?

How do you think the topic of biological invasions, specifically invasive ornamental plants, is addressed on media, educational programs and government/non-government projects?

In your opinion, what are the needs of research in this field, specifically related to the ornamental sector in your territory and region?

Can you give us your thoughts about what would be the spaces or faces of the invasion phenomenon where regional collaboration is necessary? (*e.g.* training, exchange of experts, regional database maintenance, information sharing, risk assessment, border control).

We are now finalising the interview. Thank you very much for your time and energy and for being so keen to have this conversation with me. I really appreciated it!

Would you like to know the next steps and how I will analyse these interviews?

Would you like to give any other comment that you think is important given the purpose of the study, and/or suggest any other topic I should bring into the conversation?

Can you please suggest any colleague you think will be good for me to interview and give me her/his contact details.

[Pay attention to the interviewee's response and make the interaction flows with body and verbal languages.]

Have a nice rest of the day and thank you again!

SI3: Questionnaire for ornamental related industry staff

Hello/Dumelang :),

Do you know that many plants have been introduced to Southern Africa for ornamental purposes? A few of these plants, like lantana (*Lantana camara*) and water hyacinth (*Pontederia crassipes*), have escaped from gardening and spread massively across the region, having environmental and socio-economic impacts that vary depending on the context. We are now conducting some research to better understand the relationship between the ornamental sector and the spread of introduced ornamental plants.

If you run and/or work in a business within the ornamental/horticultural sector in Botswana/Namibia/Zimbabwe, we would appreciate it if you could take 25-30 minutes of your valuable time to answer this survey.

A o itse ka matlhare a tsisitswêng mo Borwajwa Aforika, mabaka le go kgabisa? Dikai tsa ditlhare tse, ebong lantana (*Lantana camara*) le hyacinth ya mêtsi (*Pontederia crasspies*), di falôtse tshingwana go tswa tsa tlhoga mo metsteng ka kakarêtsô. Go tlhaga sešeng ga matlhare a, go rotloêditse ditlamoragô tse di farologaneng mo itsholêlông ya ditšhaba tse di farologaneng. Ke ka mo, re êtêlêla dipatlisisô a go leka go tlhalôganya kamanô ya lephata la mekgabiso le go anamisa ga ditlhare tse di tsisitswêng.

Fa o nale kgwêbô mo lephatêng la mekgabiso/temo ya dithunya le matlhare a mekgabiso mo Botswana, re ka leboga thusô ya gagô mo nakong ya gago e maleba go araba dipotsô. Go solôfêlwa go tsaya metsotso, ê e sa feteng 25-30 go di araba.

What is the survey about?/Patlisiso ê ka eng?

We would like to know the structure and dynamics of the ornamental sector in Botswana and assess how important introduced plants are for it. Your participation is voluntary, but if you decide to take part, you will be asked a few questions about how your business operates, the characteristics of your customer base and supply chain, as well as your understanding of the behaviour of the plants you work with. If you feel more comfortable writing in Setswana, you are welcome to do so! :)

Why does your opinion matter?/Ke ka go reng karolo ya gago mo patlômaikutlô e le botlhokwa?

You are invited to participate in this study because you work in the ornamental sector in Botswana/Namibia/Zimbabwe. By sharing your experiences with us, you will be contributing to understand better the role of the ornamental sector in facilitating the spread of alien plants in Southern Africa. The analysis of the information you give will help tackle plant invasions in the sub-region while safeguarding the flourishment of the ornamental sector.

O lalêtswa go nna le seabê mo patlisô e, ka ntlha ya kamanô ya gago le go bêrêka mo lephatêng la mekgabiso mo Botswana. Tirisanô mmôgô ya gago e ka thusa ka go tlhalôganya karolô ya lephata la mekgabiso mo kanamisô ya ditlhare tlhasêlông mo borwa jwa Afrika. Tlhatlhobô ya kitsô e o tsilêng go e fa, e tla thusa go lwantsha kanamisô tlhasêlông ya ditlhare le go gola ga lephata la mekgabiso.

What do we do with your data?/Re dira eng ka data ya gago?

The survey is anonymous. At the end, there is space for you to fill non-identifiable demographic data that will help us to understand better your opinions. However, the provision of this data is optional. If after filling the survey you decide that you do not desire us to include your responses into the study, please let us know by contacting Diana (telephone: +267 75118554; what's app: +44 7312056207). The results of this study may be summarised in published articles, reports, conferences/events and educational activities. Quotes will be always made anonymous in any formal outputs.

Patlisisô ê ga edirise maina. Phêlêlông, go na le dipotsô tsa motlhôkaleina tse di ka re thusang go tlhalôganya megopolô ya gago botoka. Le fa go ntse jalo, tlamêlôya data ke ka boikgêtlêlô. Fa o ka tsaya tswhetso ya go sa batla tirisô ya dikarabô tsa gago, tsweetswee re tsibosê ka go itebaganya le Diana (mogala: +267 75118554; whatsapp: +44 7312056207). Di karabô tsa gago di ka sobokwa mo dikgatisông, dipegô, dikôpanô le ditirô tsa thutô. Tirisô ya go nopôla kitsô go tswa mo patlisô e, di ka iriswa mme go sa itsiwe maina.

About us/Mabapi le rona

My name is Diana, a postgraduate researcher from Coventry University (England) currently doing a research internship in the Department of Soil and Crop Sciences at the Botswana University of Agriculture and Natural Resources. This study is part of my doctorate

project, which is supervised by a team of researchers from the universities of Coventry (England), Stellenbosch (South Africa) and Gaborone (Botswana).

Leina lame ke Diana, moithuti wa dithutô tsa morago, mmatlisisi go tswa Coventry University (England) mo patlisông ê e dirwang mo lenanêông a go ithutêla tirô mo tirisong, mo lephatêng la thutô ê e tsenêlêtseng ka sejwalô ko BUAN (Botswana University of Agriculture and Natural Resources). Patlisisô e ke ntlhangwe ya thutô yaaka e tswêlêtseng (doctorate project), e mo tlhôkômêlông ya setlhôpha sa dipatlisô, go tswa mmadikolo wa Coventry (England), Stellenbosch (South Africa) le Gaborone (Botswana).

Consent

If you decide to take part, please confirm below if you are comfortable with the following/ Fa o ka tsaya kgwetlhô ya go tswêlêla, tsweetswee rurifatsa fa o sa kgopisiwe ke mo go latêlang:

Please select at least 3 answer(s):

I understand my participation is voluntary and that I am free to withdraw my data, without giving a reason, by contacting the lead researcher at any time/ Ke a tlhalôganya gore ga ke patêlelwe go tsaya karolô mo patlisông e, le gore ke ka ntsha data yaaka, ke sa fe sebaka,ka go lelêtsa mmatlisi motôna, ka nakô ngw le ngwe.

I understand that all the information I provide will be held securely and treated confidentially/Ke a tlhalôganya gore kitsô e ke e fang e tla tshwarwa sentlê le ka tsela ê e babalang sephiri.

I am happy for the information I provide to be used (anonymously) in academic papers and other formal research outputs/Ke ka itumêdisiwa ke pabalo sephiri sa karolô yaaka mo tirisong ya dipampiri tsa thutô le maphata a dipatlisisô tse di maleba.

I am happy for the lead researcher, Diana, to save my contact details for further follow-up/Ke ka itumêlafa mmatlisi wa maêmô a ntlha, e bong Diana, a ka sala le megala yaaka go ka ntswhara mabapi le patlisô tse di tswêlêtseng fa go ka tlhokêga.

Questionnaire

Section 1: Business operation

In this first section, we would like to understand how your business (or the business you work in) operates/ Mo karolông ya ntlha, reka rata go tlhaloganya ka kgwebo ya gago (kgotsa e o bêrêkang ko go one).

Question 1: What role (s) do you play in thebusiness you represent?/ Seabê sa gago mo kgwebo e o buang ka yônê keng?

Question 2: If you were to sell this business to costumers, how would you describe it? Please, write no more than three lines/ Fa o ne o ka rêkisa kgwêbô ya gagô (kgotsa ê o bêrêkang mo go yônê) o ka e tlhalosêtsa barêki ba gago jang? Tsweetswee kwala diêle tse tharô.

Question 3: How long has the business operated?/Kgwebo ya gago e nale dingwaga tse kae?

Question 4: How long have you had (and/or workedin) this business?/O nale dingwaga tse kae o bêrêka mo kgwêbông e?

Question 5: Do you have anyformal qualification in the field of horticulture/gardening/landscaping?/ A o nale borutêgi jo bo tlhômameng mo lephata la temo ya maungo le merôgô/di tshingwana/ kgwebo ya go tsôsôlôsa bontlê jwa naga kgotsa tikologô?

If yes, please, name it/Fa karabô e le ee, ke ka eng?

If no, please briefly describe (in no more than two lines) how you got the experience/Fa karabô e le nnyaa, tsweetswee tlhalôsa ka bokhutswane tsela ê o e tsêreng go filtha o nale kitsô e o naleng yônê?

Question 6: With respect to similar businesses in Botswana/Namibia/Zimbabwe, do you consider this business to be:/Kamanô le dikwgêbô tse di tshwanang mo Botswana, a o akanya gore kgwebo ya gago (kgotsa e o berekang ko go yônê) e:

__small/nyenyane __medium/selekanyô se se fa gare __ large/tôna __ do not know/ga ke itse

If you know, please give an estimate of the staff number, the size of the establishment in square meters and/or the number of branches/O ka fa palo ya babêrêki, le fa o e akanyêtsa, bo tôna jwa madirêlô mo disekweremetara.

Question 7: Which village/town/city (s/es) of Botswana/Namibia/Zimbabwe is this business located?/ Kgwêbô ya gagô (kgotsa ko o bêrêkang têng) e fitlhêlwa mo metseng/ torôpô efe?

Question 8: Could you give details of the business' customer base?/Mothêô wa barêki jwa kgwêbô ke bo mang?

Please, rank the types of customers from the least (1) important to the most adding 1 as importance increases (e.g. 2, 3, 4...). If you know, give an estimate of the percentage each of them represent across the customer base/Tsweetswee tlhôpha tatêlanô a maêmô a barêki go ya ka selekanyô sa botlhôkwa, kamanô le kgwêbô, (1) e le kamanô le bonyenyane.

	**Rank**	**Percentage across the customer base**
domestic/home gardens (public)/ ditshingwana tsa mo lapêng (tsa setšaba)		
commercial landscapers/dikwgêbô tsa naga		
retailers/barêkisi		
nurseries/majalêlô a ditlhatshana tse di rêkisang		
businesses from other sectors/dikwgêbô (go tswa mo)		

If the business has other types of customers not mentioned above, please outline and rank them/Fa tse dingwe Tsweetswee kwala mefuta e mengwê ya barêki le maêmô a bone):

Question 9: Where do the customers come from? Please tick as many as apply to you and state the name of the area you refer to, if you know. Please, rank them importance to your business from the least important(1) to the most/ Barêki ba gago ba tswa kae? Tsweetswee tlhôpha tsotlhê tse di go lebaganeng, le maina a lefelo e o e rayang, fa o e itse.

Tsweetswee di kwala ka go latêlang a maêmô a tsonê, go ya ka botlhôkwa jwa maêmô a ntlha go fitlha a maêmô a bofêlô.

__same village/town/city as where the business is located/Ba tswa mo motse/ torôpô e kgwêbô ya gagô e fitlhêlwang têng

__ different village/town/city as where the business is located but in the same district/region/province/ba tswa mo motse/torôpô e kgwêbô ya gago e sa fitlhêlwang têng mme ba le mo kgaolông e le ngwe

__ different village/town as where the business is located in a different district/ba tswa mo motse/torôpô e kgwêbô ya gago e sa fitlhêlwang têng ba le mo kgaolông ê e farologaneng

__ village/town/city in neighbouring countries/motse/torôpô mo mafatsheng a a bapilêng

__ other/ba tswa mo mafelong a a sele __ do not know/ga ke itse

If you chose different village/town/city(s) as where the business is located, please name it/Fa ba tswa mo motse/torôpô e kgwêbô ya gago e sa fitlhêlwang têng, tsweetswee tlhalôsa:

If you selected Other, please specify/ Fa ba tswa mo mafelong a a sele, tsweetswee tlhalôsa:

Section 2: Factors influencing the business' operation

In this section, we would like to understand the factors influencing the business' operation and the sources of materials/Mo karolông e, re batla go tlhaloganya dintlha tse di tlhotlhelêtsang ditirô tswa kgwebo le motswêdi wa didirisiwa.

Question 10: What are the top 5 plants most popular amongst the business' customers that are sold/used the most? Please rank them from the least (1) to the most. You can use either common names, scientific names or a description of the plant/ Ditlhare tse tlhano tse di ratiwang thata go fetisa tse dingwe ke tse fe' tse di rêkisang ke barêki? Tsweetswee di kwalê ka go latêlana ka tse di rekwang thata go ya ka tse di sa rêkiwang (1). O ka dirisa maina a tlwaêtsweng, kgotsa maina a sense (scientific names) kgotsa tlhalosô jwa setlhare.

Please state two reasons why you think these plants are the most popular amongst the costumers/Tsweetswee fa mabaka a le mabêdi go tlhalôsa se o akanyang se dirang gore ditlhare tse di ratiwe ke barêki.

Question 11: In general, what do you think are the main factors influencing plant purchase/use amongst customers? Please, assign a value to each of them (0=no

importance, 1=minor important, 2=medium importance, 3=important, 4=very important, 5= do not know)/Ka kakarêtsô fêla, ke eng se o akanyang se nale thotloetsô mo thêkisô ya ditlhare mo barêki ba gago? Tsweetswee supa boleng jwa tsonê tsotlhê(0=ga gona mosola, 1=botlhôkwa jo bo ko tlase, 2=botlhôkwa jo bo fagare, 3= botlhôkwa, 4=botlhôkwa jo bo ko godimô, 5= ga ke itse).

	**0**	**1**	**2**	**3**	**4**	**5**
Trends/fashions in the specialised clubs/groups/Bošeng/di ratiwa ka nakô nngwe lekôkô lê le itebagantseng ya di rata						
Offers from retailers/nurseries/Phokotso ya tlhôtlhwa						
Ease of propagation/Setlhare se kgôna go ikôkêtsa motlhofo						
Ease of maintenance/Di motlhofo go tlhôkômêla						
Growth speed/Lôbelô jwa go gola						
Water use/Tirisô ya mêtsi						
Utilitarian value (e.g., firewood, food, medicinal)/Mosola mongwe (e.g., dikgong, dijô, melemô)						
Provision of protection/privacy/Pabalêlo sephiri tse di dirwang ke setlhare						
Shade provision/Moriti wa tsonê						
Beauty/decorative value/Boleng jwa go kgabisa/bontlê jwa tsonê						
Presence and duration of flowers/blooming/Nako le sebaka sa dithunya/go thunya						
Foliage and flower colours/Ditlhare le mebala a dithunya						
Fragance/Menko a tsonê						
Wildlife attraction (e.g., birds, bees)/Diphôlôgôlô tsa naga (dinyônyane, dinôtshi)						
Traditions/Ngwaô						
Spiritual and/or religious value/ Boleng jwa bodumêdi kgotsa bomoya						
Memories attached to the plant						
Price						

If you think of other criteria, please outline them and state their importance/Mabaka a mangwe:

Question 12: On yourside, which criteria does the business use to select the plants you sell/use? Please, assign a value to each of them (0=no importance, 1=minor important, 2=medium importance, 3=important, 4=very important, 5= do not know)/ O dirisa mabaka afe go tlhôpha ditlhare tse o di dirisang/rêkisang? Tsweetswee supa boleng jwa tsonê tsotlhê(0=ga gona mosola, 1=botlhôkwa jo bo ko tlase, 2=botlhôkwa jo bo fagare, 3=botlhôkwa, 4=botlhôkwa jo bo ko godimô, 5= ga ke itse).

	**0**	**1**	**2**	**3**	**4**	**5**
Demand from customers/Letlhôkô la barêki						
Ease of propagation/Setlhare se kgôna go ikôkêtsa motlhofo						
Ease of maintenance/Di motlhofo go tlhôkômêla						
Trends/fashions in the specialised clubs/groups/Thotloetsô ya di magazine/mafokô						
Offers from providers/Phokotso ya tlhôtlhwa go tswa mothêô						
Costs/Tlhwatlhwa						
Water use/Tirisô ya mêtsi						
Geographical origin						

If you think of other criteria, please outline them and state their importance/Mabaka a mangwe:

Question 13: Could you say where the business sources the plants from?/Ditlhare tsa gago di tswa mo mothêông ofe?

If yes, please rank them in order of importance to the business, being 1 the least importantand adding 1 as importance increases (e.g., 2, 3, 4...). If you know, give an estimate of the percentage of material sourced from each of them/Tsweetswee, fa o itse, fa kakanyêtsô ya palô ya didirisiwang e o e dirisang go tswa ko ngwe le ngwe ya tsone. Supa boleng jwa tsone tsotlhê mo kgwêbông ya gago, 1 e le tse di naleng boleng jo bo ko tlase.

	Percentage of sourced material	Rank
in-house propagation/go ikôkêtsa ga matlhare a fitlhêlwang mo kgwêbông		
supply nurseries/motheo wa majalêlô a ditlhatshana tse di rêkisiwang		
retailer nurseries/majalêlô a ditlhatshana tse di rêkisiwang		
other private/informal suppliers/ moabi wa semuso/o e seng wa semuso		

If the business uses other sources not mentioned above, please outline and give percentage and/or rank them/ mabaka a mangwe, tsweetswee fa sebaka le maêmô a sonê:

Question 14: Where are the business' suppliers located? Tick as many as apply to the business, and state the name of the area you refer to, if you know. Please, rank them importance to your business, starting with 1 as the least important/Fa o nale moabi, o fitlhêlwa kae? Tlhôpha tsotlhê tse di go amang le lefelo le ole rayang, fa o itse. Supa boleng jwa tsone tsotlhê mo kgwêbông ya gago, (1) e le tse di naleng boleng jo boko tlase.

__same village/town/city as where the business is located/Ba tswa mo motse/ torôpô e kgwêbô ya gagô e fitlhêlwang têng

__ different village/town/city as where the business is located but in the same district/region/province/ba tswa mo motse/torôpô e kgwêbô ya gago e sa fitlhêlwang têng mme ba le mo kgaolông e le ngwe

__ different village/town as where the business is located in a different district/ba tswa mo motse/torôpô e kgwêbô ya gago e sa fitlhêlwang têng ba le mo kgaolông ê e farologaneng

__ village/town/city in neighbouring countries/motse/torôpô mo mafatsheng a a bapilêng

__ other/ba tswa mo mafelong a a sele __ do not know/ga ke itse

Question 15: Please, tell us the methods used to manage the plant waste resulted from the business' activities. Tick as many as apply to this case/Tsweetswee retsibose ka ditsela tse o di dirisang go latlha malele a a dirwang ke kgwebo ya gago. Tlhôpha tsotlhê tse di go lebaganeng.

__in site composting/di dirisiwa go ira motshetêlô mo lefelo la kgwebo

__off-site composting (e.g., other business, communal site, farm)/di dirisiwa go ira motshetêlô kontlê ga lefelo la kgwebo (e.g., dikgwebo tse dingwe, lefelo la setšaba, tshimô)

__waste collection by external partner/ matlakala a olêlwang ke dikgwebo tse dingwe

__ organic waste collection by external partner/matlakala a tswang mo ditsheding a olêlwang ke dikgwebo tse dingwe

__ landfill dumping/Mafelô a a kgêthêgileng a go katêlwa ga matlakala __ incineration/di a fisiwa

__ dumping in the bush/go di latlha mo sekgwa __ do not know/ga ke itse

If you selected Other, please specify/ ditsela tse dingwe, tlhalôsa:

Question 16: Does the business provide information about the plants and propagules [seeds, bulb, roots] it commercializes and/or uses in its services?/A o naya kitsô ka ditlhare le dira tsa tsone [seeds, bulb, roots] tse o di rêkisang/di dirisang mo kgwebo ya gago?

If yes, how does the business provide it?/Fa karabô e le ee, o aba kitsô e jang?

__ labels and/or leaflets/ka matshwaô __ verbally/ka puô __ other

If you selected Other, please specify/ditsela tse dingwe, tlhalôsa:

Question 17: What kind of information does the business provide to the customers? Please tick as many as apply to this case/Fa go kgônala, o abêla barêkisi ba gagô kitsô wa mofuta ofe? Tsweetswee tswhaya tsotlhê tse di go amang.

__ species and/or variety name/setlhare le leina la mofuta

__ geographical origin/lefelo le di fitlhelwang têng __ other

__ maintenance and growing requirements/tlhôkômêlô ya tsone/mo di go tlhôkang go gola

__ storage requirements (in the case of seeds, bulbs or roots)/mabêêlô a di a tlhôkang (kamanô le peo, digwere kgotsa medi)

If you selected Other, please specify/kitsô e farologaneng, tlhalôsa:

Section 3: Plant biogeography, behaviour and impact

In this third section, I would like to learn of your understanding of the biogeographical origin, behaviour and impact of plants within the business and beyond it.

Mo karolô e ya boraro, re ka rat fao ka arogana kitsô ya gagô le lefelô le di tlhôgilêng teng, boitshwarô le thotloetsô mo kgwêbô ya gagô le leatherka kakarêtsô.

Question 18: Please, tick the combinations of terms you are familiar with/Ke eng se so se tlhalôganya ka ditlhare tsa motswasele (non-indigenous, exotic, or non-native), native (or indigenous) plant, and invasive plant? Please, give a short definition of the term(s).

__ alien (exotic, non-native, non- indigenous)/motswakwa, native indigenous)/tlholêgô, invasive/ tlhasêlô

__ alien (exotic, non-native, non- indigenous)/motswakwa, native indigenous plant/tlholêgô

__ alien (exotic, non-native, non- indigenous)/motswakwa, invasive plant/tlhasêlô

__ native (indigenous) plant/tlholêgô, invasive plant/ tlhasêlô __ none of them

Question 19: Are these terms relevant to the business?/A mafokô a, a nale kamanô epê le kgwêbô ya gagô? Please, explain your answer in no more than three lines/ Tsweetswee tlhalôsa karabô ya gagô mo maêle a le mararo.

Question 20: Please, mention examples of alien plants (up to 5) and native plants (up to 5) the business commercializes/uses. You can use common or scientific names or a description of the plant/Fa go tsamaisasana, tsweetswee fa dikai tsa ditlhare tsa tlhasêlô (tse tlhanô) le ditlhare tsa tlholêgô (tse tlhanô) tse o di rêkisang/di dirisang. O ka dirisa maina a tlwaêtsweng, maina a maranyane kgotsa tlhalosô ya setlhare.

Question 21: Thinking beyond the business, are you aware of any plants considered invasive in Botswana?/ Go akanyêtsa kontlê ga kgwebo ya gago, a o nale kitsô ka matlhare a tlhasêlô mo Botswana?

Question 22: How have you found out about invasive plants in Botswana? Please tick as many as apply to you/O ne wa tla go nna le tshedimosô ka ditlhare tsa tlhasêlô mo Botswana? Tsweetswee tlhôpha tse di go amang.

__ newspapers/pampiri ya dikgang __ television/thelebišene __ magazines

__ radio/seromamôwa __ training, course/thulaganyô ya go rutiwa __ social media

__ specialized books/dibuka tse di itebagantsêng __ internet/dipatlisisô tsa enthanête

__ formal education __ academic publications

__ own experience/boitêmogêlô jwa gagô __ word of mouth from family/friends/neighbours

__ word of mouth from staff from the environmental agency/go bolêlêla batho ga babêrêki ba lephata la tikologô

__ word of mouth from academics/go bolêlêla batho go tswa dithutêgô

__ word of mouth from community members/go bolêlêla batho ga batho ba setšaba

__ do not remember/ga ke gopotse __ other

If you selected Other, please specify/kitsô e farologaneng, tlhalôsa:

Question 22: Please give up to 5 examples of these invasive plants. You can use common or scientific names or a description of the plant. Please, state whether the business has used and/or commercialized them at some point/Tsweetswee aba dikai tse tlhanô tsa ditlhare tse. O ka dirisa maina a tlwaêtsweng, maina a maranyane kgotsa tlhalosô ya setlhare. Tsweetswee kwala fa o kilê wa di didrisa kgotsa o di dirisitsê mabaka le kgatô ya kgwêbô ka nakô ngwe.

Plant/setlhare	Is the business commercializing/using it currently?/A o kilê wa di rêkisa/o a di rêkisa?	Has the business commercialized/used it before?/ A o kilê wa di dirisêtsa kgato ya kgwêbô mo nako ê e fetilêng?

Question 23: Please, state the main reasons for your decision to stop stocking or keep stocking these plants/Tsweetswee fa mabaka a gago mabapi le go tsaya tswhetso ya go êmisa go rêkisa kgotsa go tswêlêla o rêkisa ditlhare tse.

Question 23: Please refer to any benefit and/or cost that the invasive plants you mentioned before have had for the business/Tsweetswee tlhalôsa dipoelo le/kgotsa ditshenyêgêlô tse di go amilêng mo botshelông jwa gago kamanô le ditlhare tsa tlhasêlô.

Plant/setlhare	Benefits/Dipoêlo	Costs/Ditshenyêgêlô

Question 24: Has the business had, or have other businesses and/or clients you know had trouble managing the spread of any plant?/A wêna kgotsa mongwe o mo itseng (dikwgêbô tse

dingwe/barêki) wa tshwaragana le mathata a go leka go êmisa kanamisô ya setlhare sengwê?

If yes, please, mention the plant (s). You can use common or scientific names or a description of the plant/ Fa karabô e le ee tsweetswee umaka setlhare se (ditlhare tse). O ka dirisa maina a tlwaêtsweng, maina a maranyane kgotsa tlhalosô ya setlhare.

Question 25: What measures has the business and/or any of your clients/partners taken in order to control the spread of the plant(s) mentioned before?/Fa go tsamaisasana, go ne ga diragala eng kgotsa ga tsewa dikgatô tse di tsêrweng go laola go anama ga semela? Tsweetswee tswhaya tsotlhê tse di go amang.

__ digging out/ka go êpolola __ pulling out/go kumola

__ do not know or do not remember/ga ke itse kgotsa ga ke gopotse __other

__applying herbicides/ka go dirisa sebolaya tlhatshana __ cutting/go kgaola

If you selected Other,please specify/ ditsela tse dingwe, tlhalôsa:

Question 26: How has the business disposed the plant waste resulted from the measures undertaken? Tick as many as apply to this case/Fa go tsamaisasana, go ne ga diragala eng ka matlakala a ditlhare, go tswamo dikgatô tse di tsêrweng?

__ in site composting/di dirisitswe go ira motshetêlô ko lefelo la kgwêbô

__ off-site composting(e.g., other business, communal site, farm)/di dirisiwa go ira motshetêlô kontlê ga lefelo la kgwebo (e.g., dikgwebo tse dingwe, lefelo la setšaba, tshimô)

__ waste collection by external partner/matlakala a olêlwang ke dikgwebo tse dingwe

__ organic waste collection by external partner/matlakala a tswang mo ditsheding a olêlwang ke di kgwebotse dingwe

__ landfill dumping/mafelô a a kgêthêgileng a go katêlwa ga matlakala

__ dumping in the bush/go di latlha mo sekgwa __ do not remember/ga ke gopotse

__ incineration/di a fisiwa __ other/ditsela tse dingwe

If you selected Other, please specify/ ditsela tse dingwe, tlhalôsa:

Question 27: How has the business found out about managing the spread of these plants? Please, tick as many as apply to this case/Fa go tsamaisasana, o fitlhêtse kae kitsô ya go laôla kanamisô ya ditlhare tse?

__ newspapers/pampiri ya dikgang __ television/thelebišene __ magazines

__ radio/seromamôwa __ training, course/thulaganyô ya go rutiwa __ social media

__ specialized books/dibuka tse di itebagantsêng __ internet/dipatlisisô tsa enthanête

__ formal education __ academic publications

__ own experience/boitêmogêlô jwa gagô __ word of mouth from family/friends/neighbours

__ word of mouth from staff from the environmental agency/go bolêlêla batho ga babêrêki ba lephata la tikologô

__ word of mouth from academics/go bolêlêla batho go tswa dithutêgô

__ word of mouth from community members/go bolêlêla batho ga batho ba setšaba

__ do not remember/ga ke gopotse __ other

If you selected Other, please specify/kitsô e farologaneng, tlhalôsa:

Question 28: If you want to share with us any challenge the business has gone through, please outline it here:

Background and demographic information

Please, tell us your age/Tsweetswee, re bolêlêla dingwaga tsa gagô:

Please, tell us your gender/Tsweetswee, re bolêlêla bong jwa gagô:

Please, tell us which ethnic group you identify with/ Tsweetswee, re bolêlêla gore o amana le morafe ofe:

Please, tell us your past profession, if applicable/Tsweetswee re bolêlêla e o kilêng wa e dira, fa go tsamaisasana:

What level have you attained in the formal education system?/O newa fêlêla kae mo maêmô a gago a dithutô?

__ primary education/sekolo se se botlana __ secondary education/sekolo se segolwane

__ diploma __ degree __ master's __ doctorate

__ no level/ga wa tsêna sekolo __ other

If you selected other, please specify/ ditsela tse dingwe, tlhalôsa:

Please, refer the business name and contact details:

Thank you for participating in this survey. We appreciate the time, energy and knowledge you have shared with us.

Re go lebogela go tsaya karolo mo tsamaisong ya patlisiso ê. Re itumelela thata nako, maatla le go abelana kitso ga gago.

SI4: Questionnaire for plant enthusiasts

Hello/Dumelang :),

Do you know that many plants have been introduced to Southern Africa for ornamental purposes? A few of these plants, like lantana (*Lantana camara*) and water hyacinth (*Pontederia crassipes*), have escaped from gardening andspread massively across the region, having environmental and socioeconomic impacts that vary depending on the context. We are now conducting some research to better understand the relationship between the ornamental sector and the spread of introduced ornamental plants.

If you have a space with plants in Botswana, we would appreciate it if you could take 25-30 minutes of your valuable time to answer this survey.

A o itse ka matlhare a tsisitswêng mo Borwajwa Aforika, mabaka le go kgabisa? Dikai tsa ditlhare tsê, ebong lantana (*Lantana camara*) le hyacinth ya mêtsi (*Pontederia crasspies*), di falôtse tshingwana go tswa tsa tlhôga mo metsteng ka kakarêtsô. Go tlhaga sešeng ga matlhare a, go rotloêditse ditlamoragô tsê di farologaneng mo itsholêlông ya ditšhaba tsê di farologaneng. Ke ka mo, re êtêlêla dipatlisisô a go leka go tlhalôganya kamanô ya lephata la mekgabisô le go anamisa ga ditlhare tse di tsisitswêng.

Fa o nale lefelô le o jwalang ditlhare mo Botswana, re ka leboga thusô ya gagô mo nakong ya gagô e maleba go araba dipotsô. Go solôfêlwa go tsaya metsotso, ê e sa feteng 25-30 go di araba.

What is the survey about?/Patlisiso ê ke ka eng?

We would like to understand how people use ornamental plants in Botswana/Namibia/Zimbabwe and what people appreciate from different types of plants. Your participation is voluntary, but if you decide to take part, we will ask you a few questions about what plants you use and what for, where you get these plants from, as well as your gardening style and understanding of plant behaviour.

If you have two different plots where you have plants —let's say one in the city and one in the village—we invite you to fill out this questionnaire twice. Each time you would think of only one of your spaces. On the other hand, if you do not have time to fill out the questionnaire twice, then we invite you do it thinking of the space you use or have lived in the most.

In addition, if you feel more comfortable writing in Setswana, you are welcome to do so! :)

Re ka rata go itse ka sebopêgô/popêgô (structure) ya lephata la mekgabisô mo Botswana. Go tsaya karolô mo patlisisông e ke ka boithatêlô jwa gagô, mme fa o ka tsaya karolô mo patlisisông ê, o ka botswa dipotsô mabapi le ditlhare tse o di dirisang, tlhôkômêlô tsa tsonê ê o dirisang, mokgwa wa go tlamêla ditlhare tsêo, ga mmogô le go tlhalôganya mekgwa ya boitshwarô jwa ditlhare tse o bêrêkang le tsonê.

Fa o nale ditsha tsê pêdi tsê o naleng ditlhare teng—sekai fa o nale setsha ko toropong le e ngwe ko motseng—re ka leboga fa o ka araba patlisiso ê gabêdi. Mme fa o sena nako ya go karaba patlisiso e gabedi, tsweetswee araba kamanô le lefelô le o tlhôlang teng gantsi kgotsa ko ô ne o ntse teng sebaka.

Fa o ikutlwa o ka araba ka phutulogô ka temo ya Setswana, o amogelwa go ka dira jalo!:)

Why does your opinion matter?/Ke ka go reng karolo ya gago mo patlômaikutlô e le botlhokwa?

You are invited to participate in this study because you have a space with plants in Botswana. By sharing your experiences with us, you will be contributing to understanding better the role of ornamental gardening in the spread of alien plants in Southern Africa. The

analysis of the information you give will help tackle plant invasions in the sub-region while contributing to more sustainable and regenerative gardening practices. Based on our results, we might be able to develop specific advice for gardeners, plant growers/sellers and garden centres.

O lalêtswa go nna le seabê mo patlisô e, ka ntlha ya kamanô ya gago le go bêrêka mo lephatêng la mekgabiso mo Botswana. Tirisanô mmôgô ya gago e ka thusa ka go tlhalôganya karolô ya lephata la mekgabiso mo kanamisô ya ditlhare tlhasêlông mo borwa jwa Afrika. Tlhatlhobô ya kitsô e o tsilêng go e fa, e ka re thusa go tlhabolola dikgakololô tse di gorogang ko batho ba ba tlhokomelang ditshingwana, bajwadi ba ditlhare/ barekisi ba ditlhare le maphata a ditshingwana.

What do we do with your data?/Re dira eng ka data ya gago?

The survey is anonymous. At the end, there is space for you to fill non-identifiable demographic data that will help us to understand better your opinions. However, the provision of this data is optional. If after filling the survey you decide that you do not desire us to include your responses into the study, please let us know by contacting Diana (telephone: +267 75118554; what's app: +44 7312056207).

The results of this study may be summarised in published articles, reports, conferences/events and educational activities. Quotes will always be made anonymous in any formal outputs.

Patlisisô ê ga e dirise maina. Phêlêlông, go na le dipotsô tsa motlhôkaleina tse di ka re thusang go tlhalôganya megopolô ya gago botoka. Le fa go ntse jalo, tlamêlô ya data ke ka boikgêtlêlô. Fao ka tsaya tswhetso ya go sa batla tirisô ya dikarabô tsa gago, tsweetswee re tsibosê ka go itebaganya le Diana (mogala: +267 75118554; whatsapp: +44 7312056207). Di karabô tsa gago di ka sobokwa mo dikgatisông, dipegô, dikôpanô le ditirô tsa thutô. Tirisô ya go nopôla kitsô go tswa mo patlisô e, di ka iriswa mme go sa itsiwe maina.

About us/Mabapi le rona

My name is Diana, a postgraduate researcher from Coventry University (England) currently doing a research internship in the Department of Crop Sciences at the Botswana University of Agriculture and Natural Resources. This study is part of my doctorate

project, which is supervised by a team of researchers from the universities of Coventry (England), Stellenbosch (South Africa) and Gaborone (Botswana).

Leina la me ke Diana, moithuti wa dithutô tsa morago, mmatlisisi go tswa Coventry University (England) mo patlisông ê e dirwang mo lenanêông a go ithutêla tirô mo tirisong, mo lephatêng la thutô ê e tsenêlêtseng ka sejwalô ko BUAN (Botswana University of Agriculture and Natural Resources). Patlisisô e ke ntlhangwe ya thutô yaaka e tswêlêtseng (doctorate project).

Consent

If you decide to take part, please confirm below if you are comfortable with the following/ Fa o ka tsaya kgwetlhô ya go tswêlêla, tsweetswee rurifatsa fa o sa kgopisiwe ke mo go latêlang:

Please select at least 3 answer(s):

I understand my participation is voluntary and that I am free to withdraw my data, without giving a reason, by contacting the lead researcher at any time/ Ke a tlhalôganya gore ga ke patêlelwe go tsaya karolô mo patlisông e, le gore ke ka ntsha data yaaka, ke sa fe sebaka,ka go lelêtsa mmatlisi motôna, ka nakô ngw le ngwe.

I understand that all the information I provide will be held securely and treated confidentially/Ke a tlhalôganya gore kitsô e ke e fang e tla tshwarwa sentlê le ka tsela ê e babalang sephiri.

I am happy for the information I provide to be used (anonymously) in academic papers and other formal research outputs/Ke ka itumêdisiwa ke pabalo sephiri sa karolô yaaka mo tirisong ya dipampiri tsa thutô le maphata a dipatlisisô tse di maleba.

I am happy for the lead researcher, Diana, to save my contact details for further follow-up/Ke ka itumêlafa mmatlisi wa maêmô a ntlha, e bong Diana, a ka sala le megala yaaka go ka ntswhara mabapi le patlisô tse di tswêlêtseng fa go ka tlhokêga.

Section 1: Your garden, your plants/Thsimo ya gago, ditlhare tsa gago

In this first section, we would like to understand what your space with plants looks like and how you maintain it/Mo karolông ya ntlha, re ka rata go tlhalôganya sebopêgô sa lefelo le o jwalang ditlhare têng,le ditsela tsê ô di tsayang go e tlhokomela.

Question 1: Which village/town/city of Botswana/Namibbia/Zimbabwe is your space with plants (e.g., garden, yard, plot, etc.) located?/Lefelo le o jwalang ditlhare (sekai tshingwana, tshimo,lelwapa) e fitlhêlwa mo metseng, torôpô ofe mo Botswana?

Question 2: Who is responsible for that space where you have plants? Please, tick as many as apply to you/Ke mang yo ô lebageneng le tirô ya maikarabêlô a tlhokomelo ya ditlhare? Tsweetswee tlhôpha tsotlhê tsê di go amang.

__ yourself/ke wena __ a hired gardener/motlhokomedi tshingwana o hirisitsweng

__ friend/tsala __ relative/lesika __ neighbour/moagisanyi

__ partner/mokapelo mo gongwe __other

If you selected other, please specify/ ditsela tse dingwe, tlhalôsa:

Question 3: For how long have you had that space and the plants in it?/O nale sebaka se se kae o le mong wa lefelo lê o naleng ditlhare têng ebile o di jwetse?

Question 4: What are your favourite plants in your space? Please mention up to five examples and say how you use them. You can use common or scientific names or a description of the plant. Each plant can have as many uses as apply to you, but make sure to add first the main use you give to the plant in your space/Ditlhare tsê o di ratang go fetisa tsê dingwe mo lefelo la gagô ke tsêfe? Tsweetswee umuka tsê tlhanô le tsela ê ô di dirisang. O ka dirisa maina a tlwaêtsweng, kgotsa maina a seanse (scientific names) kgotsa tlhalosô jwa setlhare. Setlhare sengwe le sengwe se ka na le tirisô sa sône, mme simolola ka tirisô ya tlhwatlhwa.

Uses can be:

- ornamental/ boleng jwa go kgabisa/ bontlê jwa tsonê
- medicinal/ melemô
- food/dijô tsa bathô
- fodder/dijô tsa diphôlôgôlô
- animal repellent/ go koba diphôlôgôlô
- timber, wood/ dikgong tsa go aga
- firewood/ dikgong tsa go apaya
- spiritual/ boleng jwa bodumêdi kgotsa bomoya
- religious

- windbreak/go fokôtsa phefô
- shade/moriti wa tsonê
- hedge or barrier/pabalêlo sephiri tse di dirwang ke setlhare
- cover or binder/setshwarisi
- firescreen/go hekêtsa molelô

Question 6: When you get new plants, from where you source them? Please, rank the sources you use from the least used source (1) to the most used by adding 1 as it increases the importance (e.g., 2,3,4,5...)/Ditlhare tsa gago ditswa mo mothêông ofe? Tsweetswee, supa boleng jwa mothêô wa tsone, go ya ka tirisô tsa tsonê (1) e le tse.

__garden centres/kagô ya ditshingwana __ plant nurseries/majalêlô a ditlhatshana tse di rêkisiwang

__backyard nurseries/majalêlô a ditlhatshana tse di rêkisiwang mo dijarata tsa batho

__ side of the road nurseries/barekisi mo ditseleng

__ propagation from plants in the wild [propagules: seeds, cuttings, entire specimen, bulbs]/go ikôkêtsa ga setlhare mo sekgwa (propagules: dipeo, bontlhabongwe jo bo kgaotsweng, setlhare sotlhe, segwere)

__ your own propagation [from cultivated specimens either from your garden/yard or anyone else's garden/yard]/ tsadisô ya maitirêlô (go tswa mo ditlhare tse di lemilweng ke wena kgôtsa mongwe yo ô mo itseng)

__ plant/propagule swap with friends/relatives/neighbours/social group (e.g., church)/kanamisô ya bontlhabongwe jwa setlhare sê se ikôkêtsang le masika/ditsala /phutego ya batho (sekai ko kêrêkê)

If you use other sources, please outline and rank them/Go tswa mo mafelong a a sele (tsweetswee supa maêmô):

Question 7: We would like to learn where these plants come from. Please tick as many as apply to you and specify the name of the area you referto, if you know/Re ka rata go ithuta ka motswêdi wa ditlhare tsê. Tsweetswee tlhôpha tsotlhê tse di go amang le lefelo le ole rayang,fa o itse.

__same village/town/city as where the business is located/Ba tswa mo motse/ torôpô e kgwêbô ya gagô e fitlhêlwang têng

__ different village/town/city as where the business is located but in the same district/region/province/ba tswa mo motse/torôpô e kgwêbô ya gago e sa fitlhêlwang têng mme ba le mo kgaolông e le ngwe

__ different village/town as where the business is located in a different district/ba tswa mo motse/torôpô e kgwêbô ya gago e sa fitlhêlwang têng ba le mo kgaolông ê e farologaneng

__ village/town/city in neighbouring countries/motse/torôpô mo mafatsheng a a bapilêng

__ other/ba tswa mo mafelong a a sele __ do not know/ga ke itse

If you chose different village/town/city(s) as where the business is located, please name it/Fa ba tswa mo motse/torôpô e kgwêbô ya gago e sa fitlhêlwang têng, tsweetswee tlhalôsa:

If you selected Other, please specify/ Fa ba tswa mo mafelong a a sele, tsweetswee tlhalôsa:

Question 8: Have you given away or exchanged plants or plant propagules (seeds, cuttings, entire specimen, bulbs)?/Tlhalosa dinakô tsa tiragalô ya kanamiso ya ditlhare (kgotsa bontlhabongwe jo bo ikoketsang)?

If yes, in what frequency have you given away to or exchanged plants (or propagules) with/Tlhalosa dinakô tsa tiragalô ya kanamiso ya ditlhare (kgotsa bontlhabongwe jo bo ikoketsang).

	always/dinako tsotlhê	sometimes/ dinako tsê dingwe	often/kgapetsa kgapetsa	rarely/ka sewelô	never/ka gopê
neighbours/ baagisanyi					
friends/dintsala					
family/masika					
social groups (e.g., church)/ diphutegô tsa batho (sekai ko kereke)					
charities					
schools/dikolo					

community centres					

If you have ever given away to or exchanged plants with other groups not mentioned above, please outline them and specify the frecuency:

Question 9: Please, tell us which methods you use to manage the plant waste resulting from your gardening activities. Tick as many as apply to you/Tsweetswee, re bolelle ditsela tse o di dirisang go latlha matlakala a ditlhare, go tswa mo ditirô tsa gago mo tshingwana. Tlhopa tsotlhe tse di go amang.

__ in site composting/di dirisitswe go ira motshetêlôko lefelo la kgwêbô

__ off-site composting (e.g., other business, communal site, farm)/di dirisiwa go ira motshetêlô kontlê ga lefelo la kgwebo (e.g., dikgwebo tse dingwe, lefelo la setšaba, tshimô)

__ waste collection byexternal partner/matlakala a olêlwang ke dikgwebo tse dingwe

__ organic waste collection by external partner/matlakala a tswang mo ditsheding a olêlwang ke di kgwebotse dingwe

__ landfill dumping/mafelô a a kgêthêgileng a go katêlwa ga matlakala

__ dumping in the bush/go di latlha mo sekgwa __ do not remember/ga ke gopotse

__ incineration/di a fisiwa __ other/ditsela tse dingwe

If you selected other, please specify/ ditsela tse dingwe, tlhalôsa:

Section 2: Criteria to choose plants

In this second section, we would like to understand what aspects you consider to choose plants for your space/Mo karolô e, re ka rata go tlhalôganya dintlha tsê o itebaganyang le tsonê fa o tlhopa lefelo le o jwalang mo go yonê.

Question 10: Which of these criteria you consider to choose a plant for your space? Please, assign a value to each of them (0=do not know, 1=not important, 2=minor important, 3=medium important, 4=important, 5=very important)/O dirisa mabaka afe a botlhokwa go tlhôpha lefelo le o jwalang ditlhare teng? Tsweetswee supa boleng jwa tsonê tsotlhê(0=ga gona mosola, 1=botlhôkwa jo bo ko tlase, 2=botlhôkwa jo bo fagare, 3=botlhôkwa, 4=botlhôkwa jo bo ko godimô, 5= botlhôkwa jo bo ko godimô thata).

	0	1	2	3	4	5
Trends/fashions in the specialised clubs/groups/Bošeng/di ratiwa ka nakô nngwe lekôkô lê le itebagantseng ya di rata						
Offers from retailers/nurseries/Phokotso ya tlhôtlhwa						
Ease of propagation/Setlhare se kgôna go ikôkêtsa motlhofo						
Ease of maintenance/Di motlhofo go tlhôkômêla						
Growth speed/Lôbelô jwa go gola						
Water use/Tirisô ya mêtsi						
Utilitarian value (e.g., firewood, food, medicinal)/Mosola mongwe (e.g., dikgong, dijô, melemô)						
Provision of protection/privacy/Pabalêlo sephiri tse di dirwang ke setlhare						
Shade provision/Moriti wa tsonê						
Beauty/decorative value/Boleng jwa go kgabisa/bontlê jwa tsonê						
Presence and duration of flowers/blooming/Nako le sebaka sa dithunya/go thunya						
Foliage and flower colours/Ditlhare le mebala a dithunya						
Fragance/Menko a tsonê						
Wildlife attraction (e.g., birds, bees)/Diphôlôgôlô tsa naga (dinyônyane, dinôtshi)						
Traditions/Ngwaô						
Spiritual and/or religious value/ Boleng jwa bodumêdi kgotsa bomoya						
Memories attached to the plant						
Price						

If you use other criteria, please outline and rank them/Mabaka a mangwe:

Question 11: Which plants would you like to grow or have? Please, mention up to five examples and say in one or two words the main function the plant would have in your space (e.g., rose–ornamental). You can use common or scientific names, or a description of the plant/O ka rata go godisa kgotsa go nna le ditlhare tsefe? Tsweetswee umaka dikai, e ka nna tse tlhanô le tlhalosô ya tirisô ê e tsileng go go tswela môsola mo lefelo la gagô (sekai rose-go kgabisa). O ka dirisa maina a tlwaêtsweng, kgotsa maina a sense (scientific names) kgotsa tlhalosô jwa setlhare.

Question 12: What do you think are the most popular and most often grown plants in Botswana/Namibia/Zimbabwe? Please give up to five examples. You can use common or scientific names or a description of the plant. Please, say in one or two words why you think they are the most popular and often grown in Botswana/ Namibia/Zimbabwe.

Go ya ka wena ditlhare tse di fithelwang/jalwang gantsi mo Botswana ke tsefe? Tsweetswee umaka dikai, e ka nna tse tlhanô le tlhalosô ka mafoko a mabedi ya gore ke eng di ratiwa mo Botswana. O ka dirisa maina a tlwaêtsweng, kgotsa maina a sense (scientific names) kgotsa tlhalosô jwa setlhare.

Section 3: Plant origin, behaviour and impact

In this third section, I would like you to share with me your understanding of the biogeographical origin, behaviour and impact of plants within your space and beyond it/Mo karolô e ya boraro, re ka rata fa o ka arogana kitsô ya gagô le lefelô le di tlhôgilêng teng, boitshwarô le thotloetsô mo kgwêbô ya gagô le leather ka kakarêtsô.

Question 13: Please, tick the combinations of terms you are familiar with/Ke eng se so se tlhalôganya ka:

__ alien (exotic, non-native, non- indigenous)/motswakwa, native indigenous)/tlholêgô, invasive/ tlhasêlô

__ alien (exotic, non-native, non- indigenous)/motswakwa, native indigenous plant/tlholêgô

__ alien (exotic, non-native, non- indigenous)/motswakwa, invasive plant/tlhasêlô

__ native (indigenous) plant/tlholêgô, invasive plant/tlhasêlô

__ none of them

Please, give a short definition of the term(s) you are familiar with/Tsweetsweee tlhalosa mafoko ao ka bokhutswane.

Question 14: Please write examples of alien plants (up to 5) and native plants (up to 5) you are familiar with in the table below. You can use common or scientific names or a description of the plant. Please, say whether they are in your space or not and how they came into it. Use as many options as apply to you for each species/Tsweetswee fa dikai tsa ditlhare tsa tlhasêlô (tse tlhanô) le ditlhare tsa tlholêgô (tse tlhanô) tse o di mo lifelong la gago. O ka dirisa maina a tlwaêtsweng, maina a maranyane kgotsa tlhalosô ya setlhare. Tsweetswee umuka fa di le mo tshingwana ya gago le gore di gorogile jang mo lefelo le o jwalang mo go yone. Dirisa dikai tsotlhe tse di lebaganeng le ditlhare tse o di umakileng.

Options:

- It was already in the garden/yard/Ke e fitlhêtse mo lefelong
- I bought the plant/Ke tsisitse setlhare
- I bought the propagules [seeds, bulbs, roots]/Ke rekile dintlha tse di ikoketsang [seeds, bulbs, roots]
- I got the plant/propagule from someone else’s garden/yard/ Ke tsere setlhare/ ntlha ê e ikoketsang mo lefelo la motho o sele
- Noncommercial swap/ Kanamiso ê e sa dirisang madi
- It spread/s seeded in my garden/yard/Setlhare se ijwetse/ se ikanamisitse mo lefelo laaka
- I got the plant/propagule from the bush/Ke tsere setlhare/ntlha ê e ikoketsang mo sekgweng
- Other, please specify/Ditsela tse dingwe, tlhalôsa

	**Is it in your space?/A se mo lefelo la gago?**	**How it came into your space?/E tsile jang mo lefelo la**
alien plant 1		
alien plant 2		
alien plant 3		
alien plant 4		
alien plant 5		
native plant 1		

native plant 2	
native plant 3	
native plant 4	
native plant 5	

Question 15: Looking beyond your garden/yard, are you aware of any plants considered invasive in Botswana/Namibia/Zimbabwe?/A o nale kitso ka ditlhare tse go lebiwang e le tsa tlhasêlô mo Botswana?

Question 16: Please, write up to five examples of these invasive plants in the table below. You can use common or scientific names or a description of the plant. Please, say whether they are in your space or not and how they came into it. Use as many options as apply to you for each species/Tsweetswee fa dikai tsa ditlhare tsa tlhasêlô (tse tlhanô) le ditlhare tsa tlholêgô (tse tlhanô) tse o di mo lefelong la gago. O ka dirisa main a tlwaêtsweng, maina a maranyane kgotsa tlhalosô ya setlhare. Tsweetswee umuka fa di le mo tshingwana ya gago le gore di gorogile jang mo lefelo le o jwalang mo go yone. Dirisa dikai tsotlhe tse di lebaganeng le ditlhare tse o di umakileng.

Options:

- It was already in the garden/yard/Ke e fitlhêtse mo lefelong
- I bought the plant/Ke tsisitse setlhare
- I bought the propagules [seeds, bulbs, roots]/Ke rekile dintlha tse di ikoketsang [seeds, bulbs, roots]
- I got the plant/propagule from someone else's garden/yard/ Ke tsere setlhare/ ntlha ê e ikoketsang mo lefelo la motho o sele
- Noncommercial swap/ Kanamiso ê e sa dirisang madi
- It spread/s seeded in my garden/yard/Setlhare se ijwetse/ se ikanamisitse mo lefelo laaka
- I got the plant/propagule from the bush/Ke tsere setlhare/ntlha ê e ikoketsang mo sekgweng
- Other, please specify/Ditsela tse dingwe, tlhalôsa

	Is it in your space?/A se mo lefelo la gago?	How it came into your space?/E tsile jang mo lefelo la
invasive plant 1		
invasive plant 2		
invasive plant 3		
invasive plant 4		
invasive plant 5		

Question 17: How have you found out about invasive plants in Botswana/Namibia/Zimbabwe? Please, tick as many as apply to you/ O ne wa tla go nna le tshedimosô ka ditlhare tsa tlhasêlô mo Botswana? Tsweetswee tlhôpha tse di go amang.

__ newspapers/pampiri ya dikgang __ television/thelebišene __ magazines

__ radio/seromamôwa __ training, course/thulaganyô ya go rutiwa __ social media

__ specialized books/dibuka tse di itebagantsêng __ internet/dipatlisisô tsa enthanête

__ formal education __ academic publications

__ own experience/boitêmogêlô jwa gagô __ word of mouth from family/friends/neighbours

__ word of mouth from staff from the environmental agency/go bolêlêla batho ga babêrêki ba lephata la tikologô

__ word of mouth from academics/go bolêlêla batho go tswa dithutêgô

__ word of mouth from community members/go bolêlêla batho ga batho ba setšaba

__ do not remember/ga ke gopotse __ other

If you selected Other, please specify/kitsô e farologaneng, tlhalôsa:

Question 18: Please, refer to a benefit and/or negative impact that the invasive plants you mentioned before, have brought into your life in one or two words/Tsweetswee tlhalôsa dipoelo le/kgotsa ditshenyêgêlô tse di go amilêng mo botshelông jwa gago kamanô le ditlhare tsa tlhasêlô ka mafoko ka bokhutswane.

Plant/setlhare	Benefits/Dipoêlo	Costs/Ditshenyêgêlô

Question 19: Have you eradicated (or considered eradicating) any of the plants you mentioned before from your space?/A o kile wa nyeletsa (kgotsa wa nna le dikakanyo tsa go nyeletsa) ngwe ya ditlhare tse o di umakileng mo lefelo la gago?

Question 20: How have you done it (or considered doing it)? Please tick as many as apply to you/O kgonne jang go dira jalo? (kana ne o akantseng go irajang)?/Tsweetswee tswhaya tsotlhê tse di go amang.

__digging out/ ka go êpolola __pulling out/go kumola

__applying herbicides/ka go dirisa sebolaya tlhatshana __ cutting/go kgaola

__mulching/kobelo ya disalela tsa dimela mo

If you selected other, please specify/ ditsela tse dingwe, tlhalôsa:

Question 21: Have you or someone you know had trouble managing the spread of any plant in your space?/A wêna kgotsa mongwe o mo itseng, o kile wa tshwaragana le mathata a go leka go êmisa kanamisô ya setlhare sengwê.

If yes, please tell us which plant(s) has caused you trouble. You can use common or scientific names or a description of the plant/Fa karabô e le ee tsweetswee umaka setlhare se (ditlhare tse). O ka dirisa maina a tlwaêtsweng, maina a maranyane kgotsa tlhalosô ya setlhare.

Question 22: What method(s) have you or the person you know taken in order to control the spread of the plant(s) mentioned before. Please tick as many as apply to you/Fa go tsamaisasana, go ne ga diragala eng kgotsaga tsewa dikgatô tse di tsêrweng go laola go anama ga semela? Tsweetswee tswhaya tsotlhê tse di go amang.

__digging out/ ka go êpolola __pulling out/go kumola

__applying herbicides/ka go dirisa sebolaya tlhatshana __ cutting/go kgaola

__mulching/kobelo ya disalela tsa dimela mo __no method/ga go tsamaisane

If you selected other, please specify/ ditsela tse dingwe, tlhalôsa:

Question 23: How have you or the person you know disposed the plant waste resulting from the method(s) undertaken?/Fa go tsamaisasana, go ne ga diragala eng ka matlakala a ditlhare, go tswa mo dikgatô tse di tsêrweng?

__ in site composting/di dirisitswe go ira motshetêlôko lefelo la kgwêbô

__ off-site composting (e.g., other business, communal site, farm)/di dirisiwa go ira motshetêlô kontlê ga lefelo la kgwebo (e.g., dikgwebo tse dingwe, lefelo la setšaba, tshimô)

__ waste collection byexternal partner/matlakala a olêlwang ke dikgwebo tse dingwe

__ organic waste collection by external partner/matlakala a tswang mo ditsheding a olêlwang ke di kgwebotse dingwe

__ landfill dumping/mafelô a a kgêthêgileng a go katêlwa ga matlakala

__ dumping in the bush/go di latlha mo sekgwa __ do not remember/ga ke gopotse

__ incineration/di a fisiwa __ other/ditsela tse dingwe

If you selected other, please specify/ ditsela tse dingwe, tlhalôsa:

Question 24: How have you or the person you know found out about managing plant spread? Please tick as many as apply to you/ Fa go tsamaisasana, o fitlhêtse kae kitsô ya go laôla kanamisô ya ditlhare? Tlhôpha tsotlhê tse di go amang.

__ newspapers/pampiri ya dikgang __ television/thelebišene __ magazines

__ radio/seromamôwa __ training, course/thulaganyô ya go rutiwa __ social media

__ specialized books/dibuka tse di itebagantsêng __ internet/dipatlisisô tsa enthanête

__ formal education __ academic publications

__ ownexperience/boitêmogêlô jwa gagô __ word of mouth from family/friends/neighbours

__ word of mouth from staff from the environmental agency/go bolêlêla batho ga babêrêki ba lephata la tikologô

__ word of mouth from academics/go bolêlêla bathogo tswa dithutêgô

__ word of mouth from community members/go bolêlêla batho ga batho ba setšaba

__ do not remember/ga ke gopotse __ other

If you selected other, please specify/kitsô e farologaneng, tlhalôsa:

Background and demographic information

Please, tell us your age/Tsweetswee, re bolêlêla dingwaga tsa gagô:

Please, tell us your gender/Tsweetswee, re bolêlêla bong jwa gagô:

Please, tell us your profession/Tsweetswee re bolêlêla tiro ya gagô:

Please, tell us your profession, if applicable/Tsweetswee re bolêlêla e o kilêng wa e dira, fa go tsamaisasana:

What level have you attained in the formal education system?/O newa fêlêla kae mo maêmô a gago a dithutô?

__ primary education/sekolo se se botlana __ secondary education/sekolo se segolwane

__ diploma __ degree __ master's __ doctorate

__ no level/ga wa tsêna sekolo __ other

If you selected other, please specify/ ditsela tse dingwe, tlhalôsa:

Please, refer the business name and contact details:

Thank you for participating in this survey. We appreciate the time, energy and knowledge you have shared with us.

Re go lebogela go tsaya karolo mo tsamaisong ya patlisiso ê. Re itumelela thata nako, maatla le go abelana kitso ga gago.

SI5: Workshop presentation for members of the Centre for Invasion Biology at Stellenbosch University, South Africa

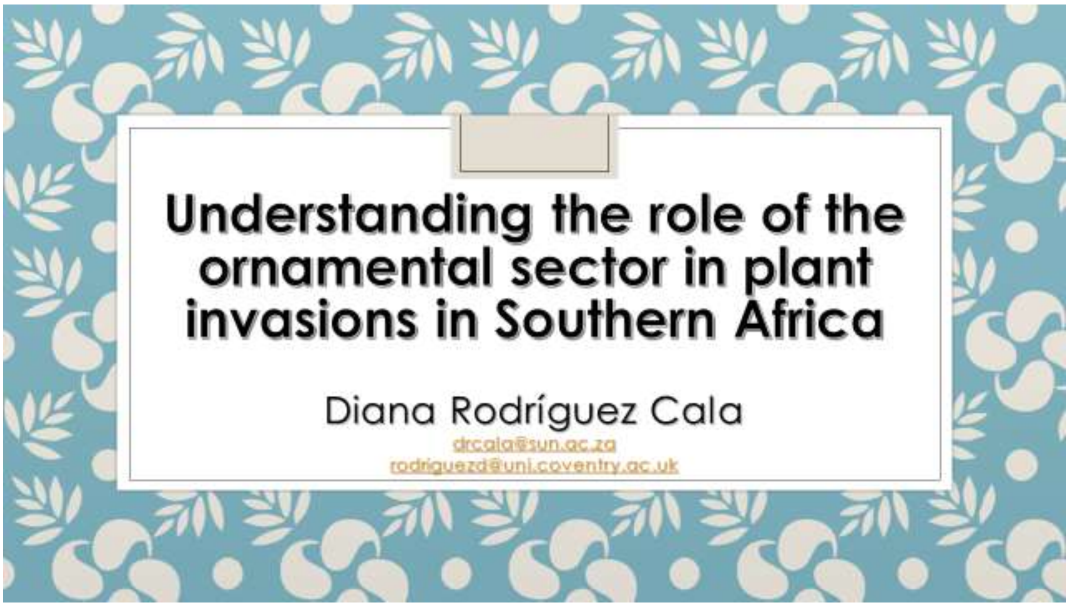

How does the ornamental sector contribute to plant invasions in Southern Africa?

- Identify the structure and dynamics of the ornamental trade/exchange of alien plants in the region
- Identify the dynamics of the use of alien ornamental plants in the region
- Assess people's perceptions of alien ornamental plants and the link between the ornamental sector and invasions in the region

The project has been divided into two phases:
a) remote-based fieldwork at a broader scale, and
b) in situ fieldwork at a narrower scale

Critical realistic and qualitative approach

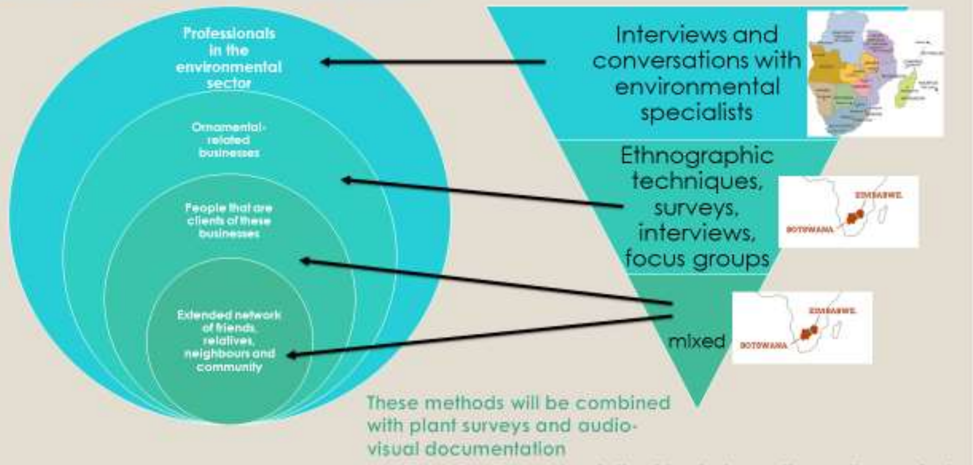

Analytical technique: thematic analysis

Ornamentals and plant invasions in Southern Africa: the environmental specialist's side of the story

What do people involved in the environmental sector in Southern Africa think about the role the ornamental sector might play in the spread of alien plants across the sub-region?

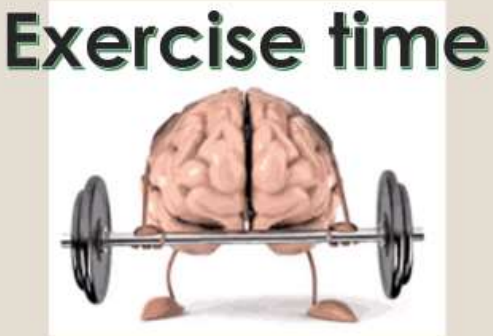

Exercise time

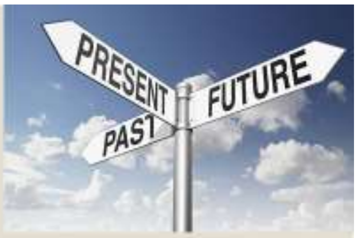

1. I will pose a question.
2. Each of you is invited to answer the question by writing and/or schematizing on the sticky notes (with your own perception in the past, present and future).
3. We will collectively debate the answers.
4. I will present what other peers have answered to this same question and other aspects derived from it.

In order to include your opinions in my research, I would like to:
Record the session
Keep the sheets you have written

Your names will be anonymised

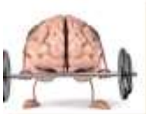

What do you think

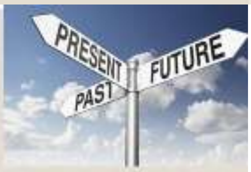

- have been (past),
- are (present), and
- might be (future)

mechanisms by which the ornamental sector influences alien plant spread in South Africa (and/or Southern Africa)?

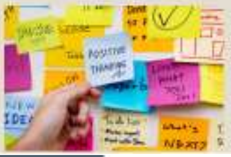

Please only one point per sticky note

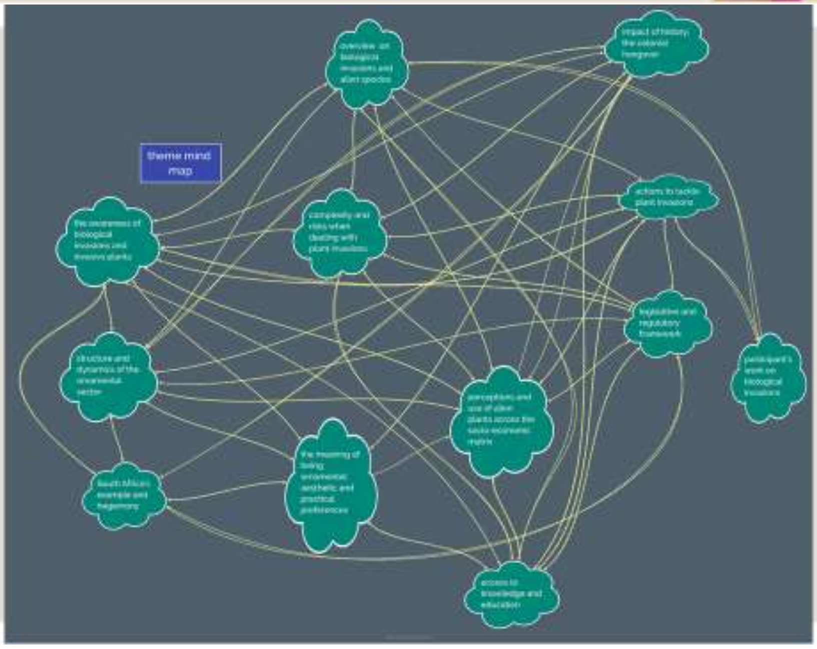

SI6: Presentation for members of the Botanical Society of Namibia

Focus group with the Botanical Society of Namibia

Links between ornamental gardening practices and alien plants in southern Africa

Diana Rodríguez-Cala[1,2,3], Jana Fried[1], John R. Wilson[4,3], Katharina Dehnen-Schmutz[1], Seoleseng O. Tshwenyane[2], Israel Legwaila[2]

[1]Centre for Agroecology, Water and Resilience, Coventry University, Coventry, UK

[2]Department of Crop and Soil Science, Botswana University of Agriculture and Natural Resources, Gaborone, Botswana

[3]Centre for Invasion Biology, Department of Botany and Zoology, Stellenbosch University, Stellenbosch, South Africa

[4]South African National Biodiversity Institute, Kirstenbosch Research Centre, Cape Town, South Africa

Corresponding author: Diana Rodríguez Cala, rodriguezd@uni.coventry.ac.uk

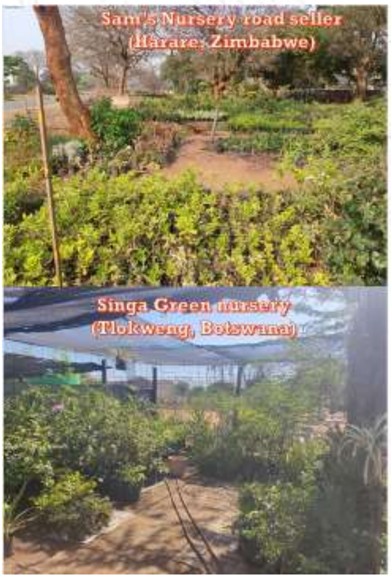

Diana Rodríguez Cala

Exploring the ornamental gardening sector in southern Africa with critical environmental lens

Department of Crop Science, Botswana University of Agriculture and Natural Resources
Centre for Agroecology, Water and Resilience, Coventry University
Centre for Invasion Biology, Stellenbosch University

Let's play a game to break the ice and warm up our brains

- We split in groups of three. For that, I will hand out pieces of paper with numbers written from 1 to 10. The persons with the same numbers will form a team.
- Each team will choose a name (be creative, please, since this will be your crowd for the whole session). You will have up to one minute to come up with an original name and let us know.
- I will reveal three statements that each team must complete in one and a half minutes. When all the statements are completed, a team representative will write them down in the flip chart I have already prepared.
- We will discuss the answers collectively to deep into the talk's core topic.

Remember you have 1:30 minutes for each statement. They will appear accordingly

One of the most popular ornamental plants in Namibia is...

One of the most weedy plants in Namibia is...

One of the most useful plants in Namibia is...

How do we have plants that are not from here?

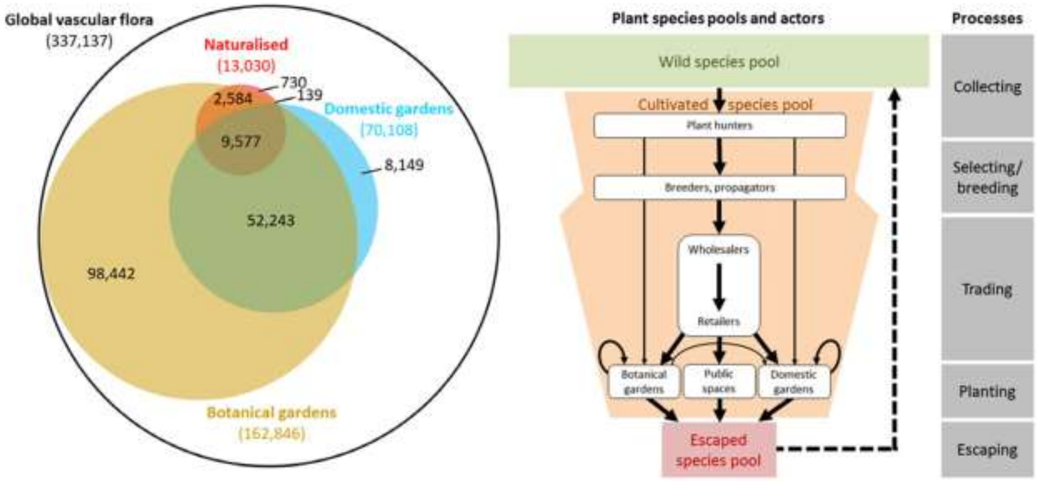

Van Kleunen *et al.* 2019

How do we have plants that are not from here?

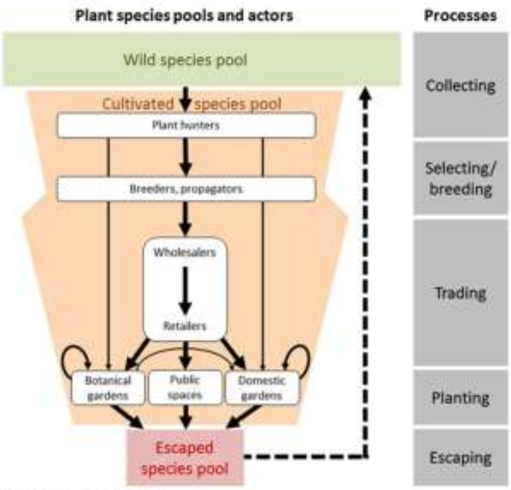

Van Kleunen *et al.* 2019

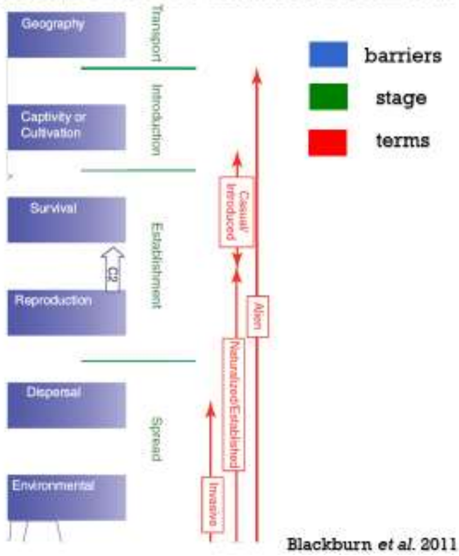

Blackburn *et al.* 2011

Percentage of exotic flora introduced for ornamentation in some southern African countries

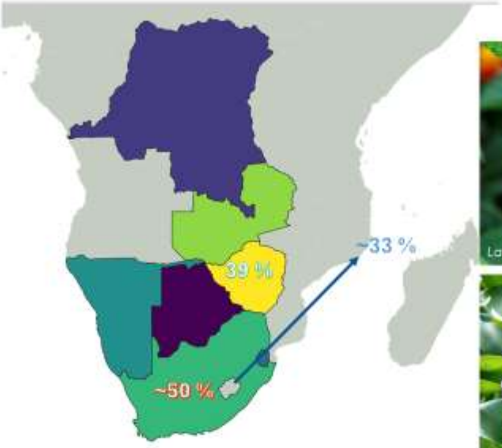

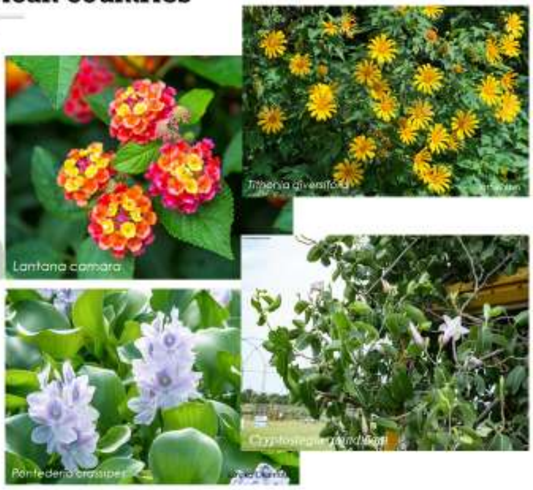

Henderson (2006); Maroyi (2017); Kobisi *et al.* (2019)

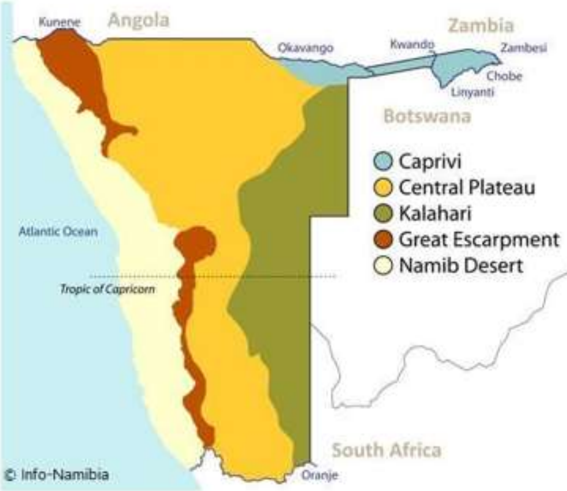

268 exotic/alien plant species

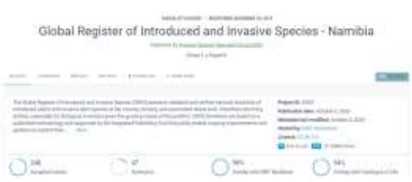

247 of which are considered invasive

Chase & Pagad (2020)

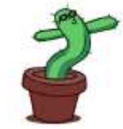

Cacti

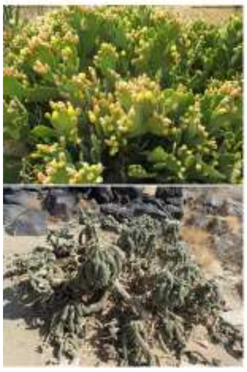

A Native range of the family Cactaceae (1922 species)

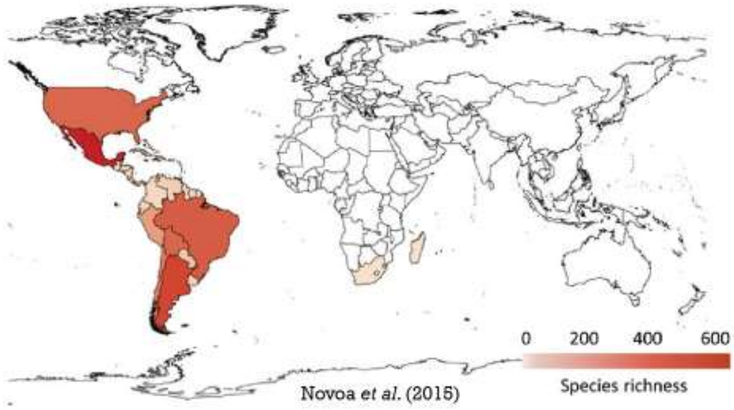

Novoa *et al.* (2015)

Exotic cacti in Namibia

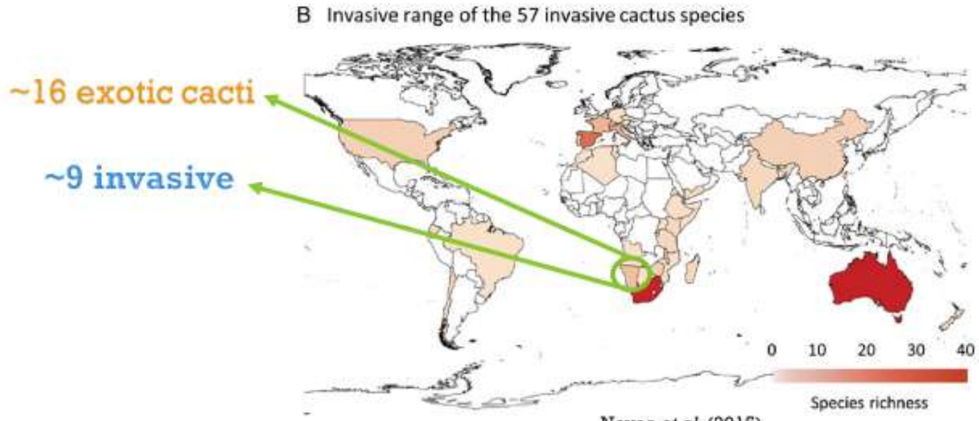

Chase & Pagad (2020)

Novoa *et al.* (2015)

Non invasive species

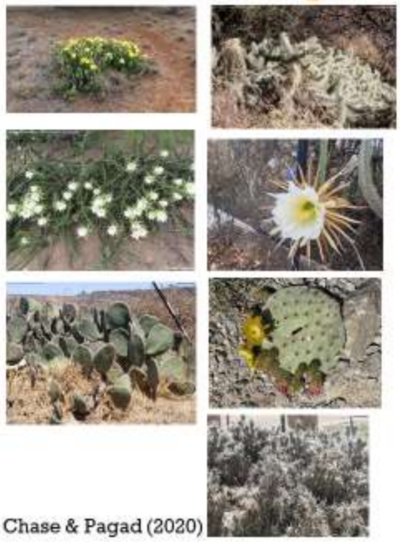

Chase & Pagad (2020)

Invasive species

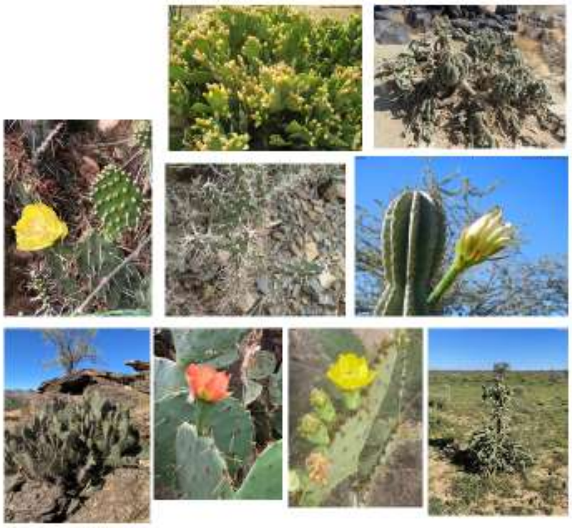

Now, let's spot patterns across these exotic cacti species

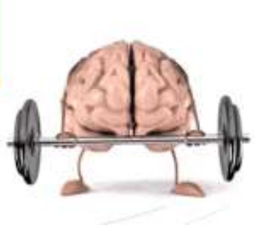

Each team will take up to 2 cacti species and describe them according to:

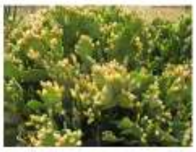

propagation traits

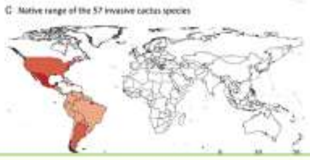

latitudinal and longitudinal range geographical similarity

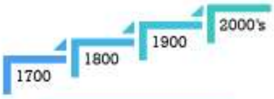

residence time

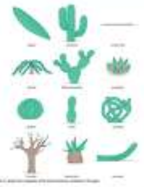

growth form

invasion status

Novoa *et al.* (2015)

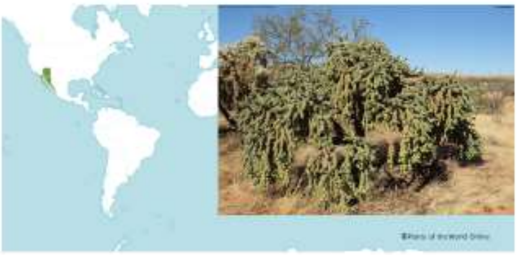

Novoa *et al.* (2015)

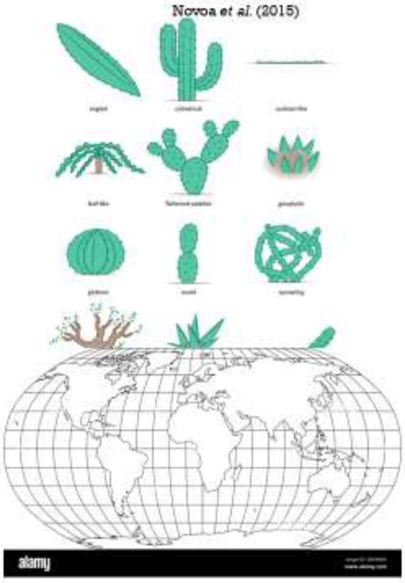

- I will provide: sheets with information about the species, a figure showing different growth forms for cacti and a blank and white world map
- You have up to 2 minutes to: highlight their range in the map and compare it with Namibia's geography, and describe the species
- We will discuss the emerging patterns collectively

Non invasive species

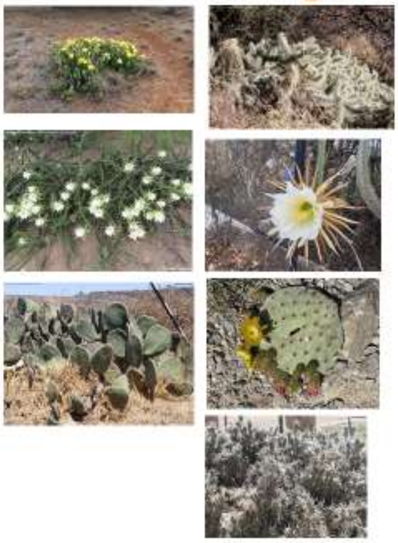

Invasive species

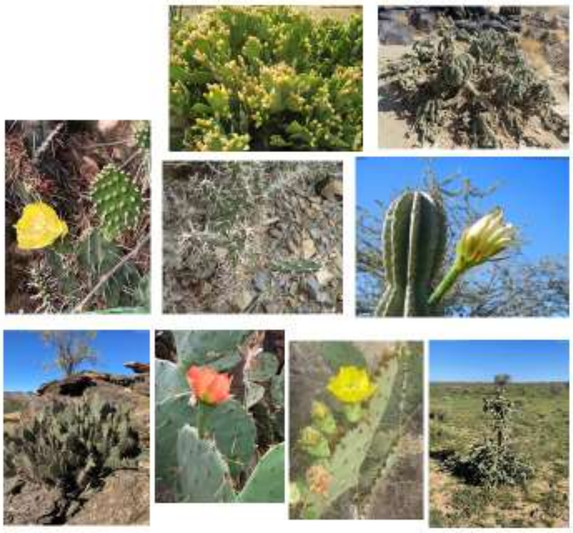

	Ovoid	Flattened-padded	Cylindrical	Sprawling	Prostrate
Vegetative propagation					
Sexual propagation					
Both types					

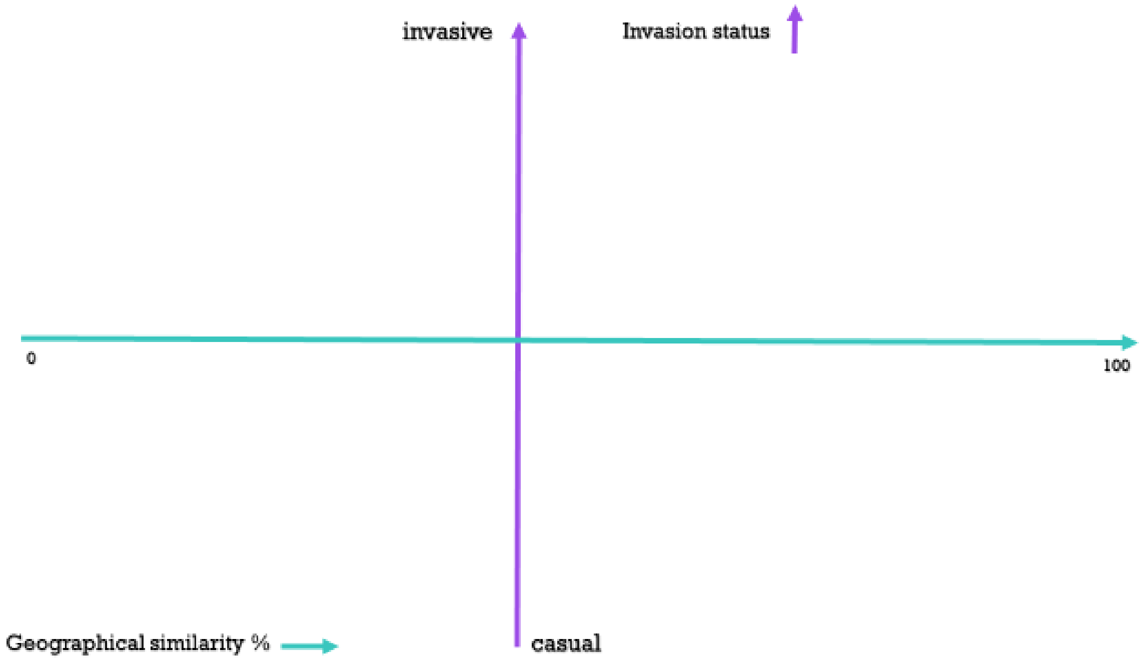

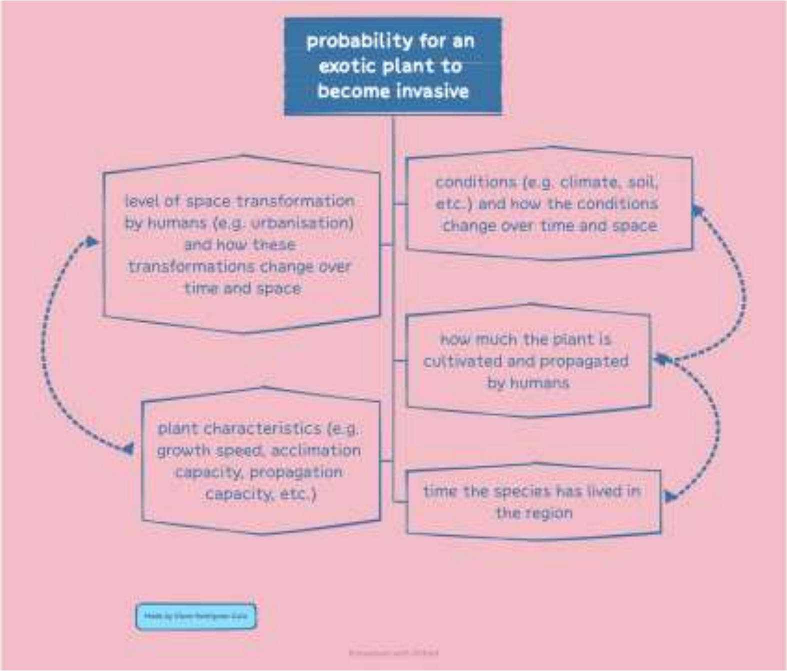

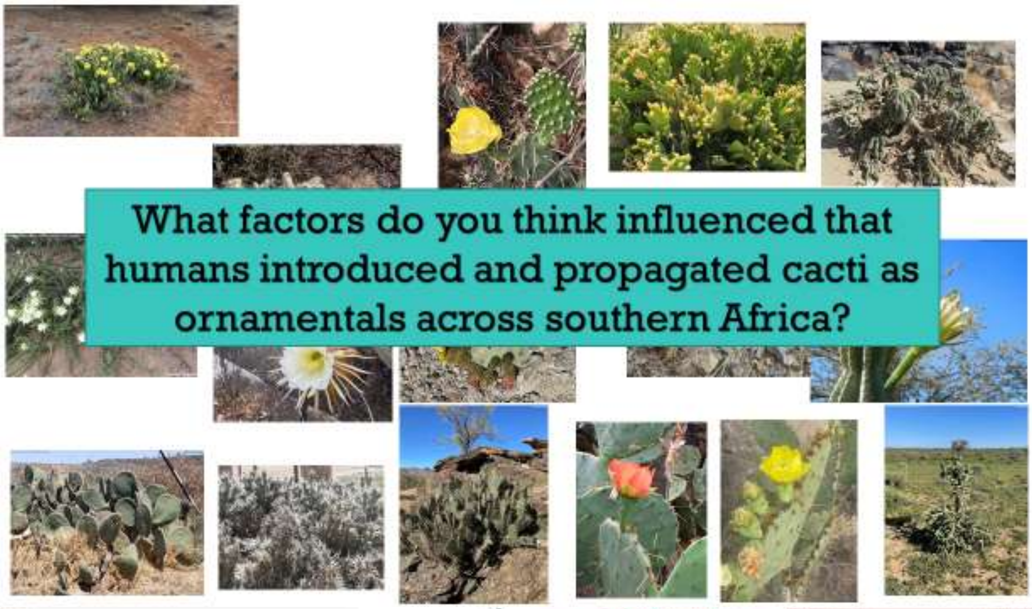

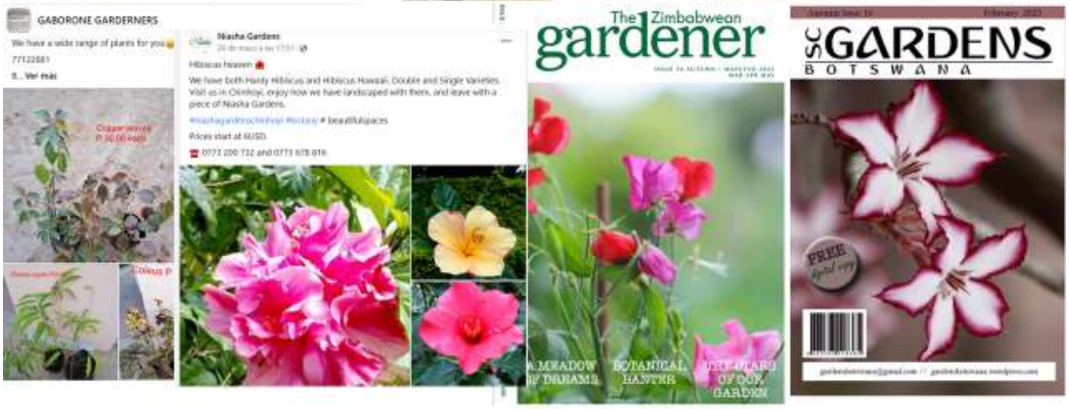

What is the current role of the ornamental sector in exotic plant spread across southern Africa?

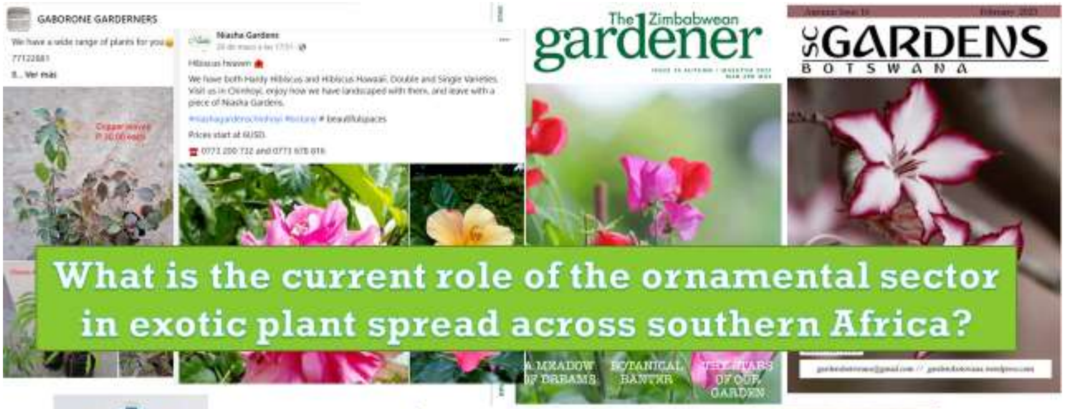

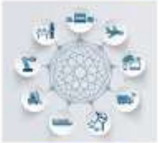
structure & dynamics

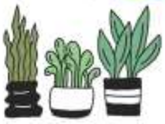
what plants are used and traded as ornamentals

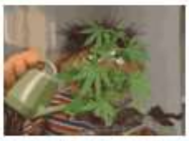
how people use and perceive ornamental plants

Since August 2021, I have been interacting with different stakeholders in southern Africa

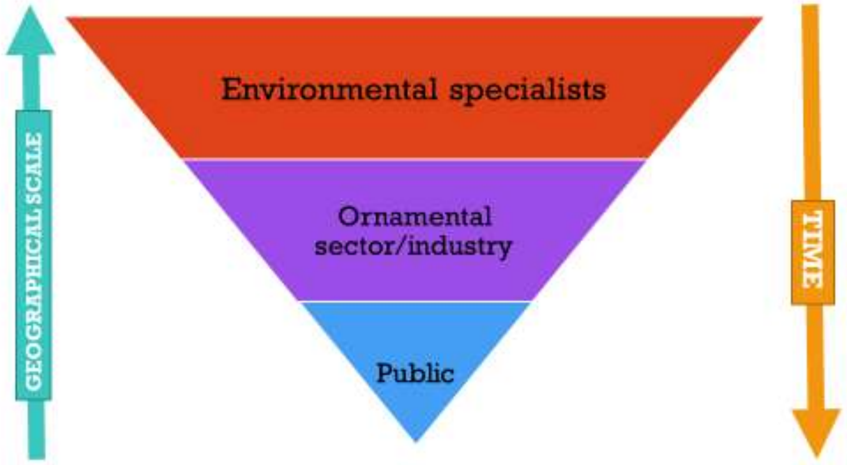

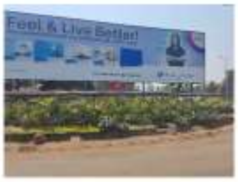

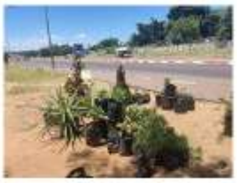

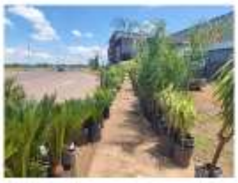

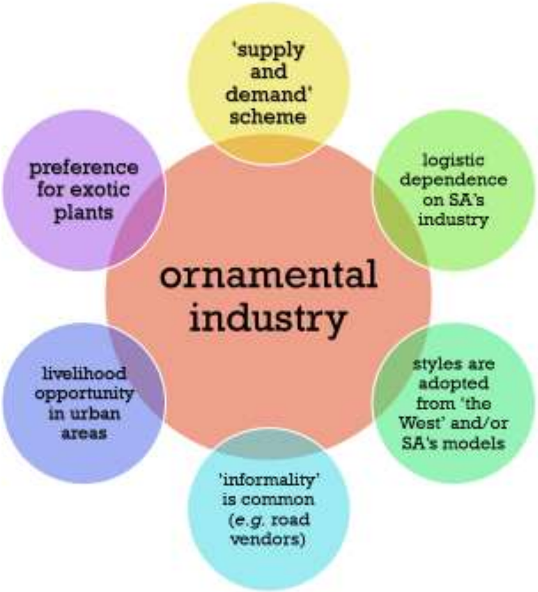

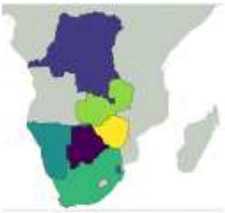

What phase am I at right now?

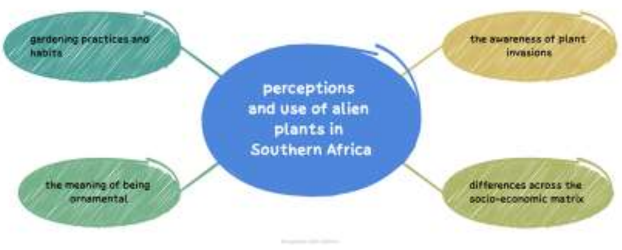

I would like to understand **how people garden** in southern Africa and **what people appreciate from different types of plants**.

I am asking questions about **what plants you use and what for, where you get these plants from**, as well as **your gardening style and understanding of plant behaviour**.

If you allow me:

I will pose a few questions that we will follow up collectively.

I will record the session to be able to catch it all for the posterior analysis.

I will provide a sheet for you to optionally fill in with demographic data (*e.g.*: age, profession, gender).

The information you give is anonymous. Its analysis **will help tackle plant invasions in the sub-region** while **contributing to more sustainable and regenerative gardening practices**. Based on our results, **we might be able to develop specific advice for the public gardeners and nurseries.**

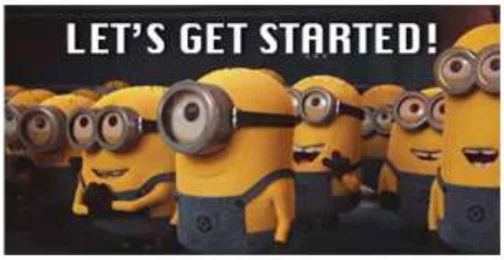

What are your favorite plants?

What about these plants you mentioned, makes them your favourite?

Where do you usually source plants from in Namibia?

When you decide to have a plant, what factors do you bear in mind?

What plants would like to have?

What challenges do you usually face to do gardening?

Let's wrap up playing another game!

JOINT PAINTING

1. Let's divide us into two groups.
2. I will give each group a sheet of paper.
3. Each person should draw whatever they feel like drawing after this session.
4. You will start with the music.
5. When I pause the music, each of you will move to the next person's space and re-start drawing.

Let's do it for a couple of times ☺ and we will be done
