# Supplementary material for: Ornamental horticulture in Southern Africa: strategic actions to address biological invasions
Source: Environ Manage. 2025 Aug 28;75(12):3203–19. doi: 10.1007/s00267-025-02241-y (PMC12575546; doi:10.1007/s00267-025-02241-y)
Supplement: Supplementary file 2 — Online Resource 2 [file 267_2025_2241_MOESM2_ESM.pdf]

Online Resource 2

Title: Ornamental horticulture in Southern Africa: strategic actions to address biological invasions

Diana Rodríguez-Cala[1,2,3], Jana Fried[1], John R. U. Wilson[4,3], Katharina Dehnen-Schmutz[1], Seoleseng O. Tshwenyane[2], Israel Legwaila[2]

[1]Centre for Agroecology, Water and Resilience, Coventry University, Coventry, UK

[2]Department of Crop and Soil Science, Botswana University of Agriculture and Natural Resources, Gaborone, Botswana

[3]Centre for Invasion Biology, Department of Botany and Zoology, Stellenbosch University, Stellenbosch, South Africa

[4]South African National Biodiversity Institute, Kirstenbosch Research Centre, Cape Town, South Africa

Corresponding author's email: Diana Rodríguez Cala, dianarodriguezcala@gmail.com/ rodriguezd@uni.coventry.ac.uk

Table 1: Laws related to managing and preventing biological invasions in Botswana, Eswatini, Namibia, the Democratic Republic of Congo (DRC), Zambia, and Zimbabwe.

Country	Law	Scope	Link
Botswana	Aquatic Weeds (Control) Act 1971	Mandates for the prevention, management and eradication of aquatic weeds.	https://www.fao.org/faolex/results/details/en/c/LEX-FAOC006484/
Botswana	Noxious Weeds Act 1916	Mandates for the eradication of weeds.	https://www.fao.org/faolex/results/details/en/c/LEX-FAOC006491/
Botswana	Noxious Weed Order 1968	List of weeds to be eradicated according to the Noxious Weed Act. It includes some aquatic weeds listed in the Aquatic Weeds Act.	https://www.fao.org/faolex/results/details/en/c/LEX-FAOC006490
Botswana	Plant Protection Act	Measures for the protection of plants from pests and diseases.	https://www.fao.org/faolex/results/details/en/c/LEX-FAOC126405/
Botswana	Forest Act 1968	Rules for the management and conservation of forest resources and for the trade of products of the forest including protected fauna and flora.	https://www.fao.org/faolex/results/details/en/c/LEX-FAOC002748/

Botswana	Wildlife Conservation and National Parks Act 1992	Framework for wildlife and national parks management.	https://www.fao.org/faolex/results/details/en/c/LEX-FAOC004728/
Botswana	Water Act 1968	Guide for the establishment of the Water Apportionment Board and the rights in respect of water and related matters.	https://www.fao.org/faolex/results/details/en/c/LEX-FAOC042103/
Eswatini	Control of Tree Planting Act, 1972	Guides the control of the planting of certain trees grown for commercial purposes in specified areas.	https://eswatinilii.org/akn/sz/act/1972/7/eng@1998-12-01
Eswatini	Environment Management Act, 2002	Framework for promoting the enhancement, protection and conservation of the environment, sustainable management of natural resources.	https://eswatinilii.org/akn/sz/act/2002/5/eng@2002-11-22
Eswatini	Flora Protection Act, 2001	Framework for protecting indigenous flora.	https://eswatinilii.org/akn/sz/act/2001/5/eng@2001-07-13
Eswatini	Forests Preservation Act	Framework for the preservation of trees and forests growing on Government land.	https://eswatinilii.org/akn/sz/act/1910/14/eng@1998-12-01
Eswatini	Grass Fires Act	Guide for consolidating the law relating to grass burning and grass fires.	https://eswatinilii.org/akn/sz/act/1955/44/eng@1998-12-01
Eswatini	Komati River Basin Water Resources Development and Utilization Act, 1992	Guidance for the implementation of the Komati River Basin Treaty.	https://eswatinilii.org/akn/sz/act/1992/6/eng@1998-12-01
Eswatini	Natural Resources Act, 1951	Framework for the conservation and improvement of the natural resources.	https://eswatinilii.org/akn/sz/act/1951/71/eng@1998-12-01
Namibia	Weeds Ordinance 19 of 1957	Mandates for the eradication of weeds.	https://www.fao.org/faolex/results/details/fr/c/LEX-FAOC188732
Namibia	Plant Quarantine Act 7 of 2008	Guide for the prevention, monitoring, control and eradication of plant pests.	https://www.fao.org/faolex/results/details/en/c/LEX-FAOC094398/
Namibia	Environmental Management Act 7 of 2007	Mandates for the protection of the environment and measures to ensure sustainable development.	https://www.fao.org/faolex/results/details/en/c/LEX-FAOC082643/
Namibia	Forest Act 12 of 2001	Mandates for the establishment of a Forestry Council and the appointment of	https://www.fao.org/faolex/results/details/en/c/LEX-FAOC046518/

		certain officials; consolidation of the laws regulating the management and use of forests and products of the forest; protection of the environment and the control and management of forest fires.	
Namibia	Nature Conservation Amendment Act 3 of 2017	Framework for the conservation and management of wildlife, regulation of fishing in inland waters, regulation of tourism in protected areas, and trade of live animals.	https://www.fao.org/faolex/results/details/en/c/LEX-FAOC188683/
Namibia	Water Resources Management Act 11 of 2013	Framework for the management and conservation of all water resources.	https://www.fao.org/faolex/results/details/en/c/LEX-FAOC134273/
DRC	Loi n° 11/022 du 24 décembre 2011 portant principes fondamentaux relatifs a l'agriculture	Mandates for the practice of agriculture.	https://www.fao.org/faolex/results/details/en/c/LEX-FAOC109785/
DRC	Loi n°11/009 du 09 juillet 2011 portant principes fondamentaux de gestion de l'environnement	Framework for the protection of the environment and measures to ensure sustainable development.	https://medd.gouv.cd/loi-n-11-009-du-09-juillet-2011-portant-principes-fondamentaux-relatifs-a-la-protection-de-lenvironnement/
DRC	Loi n° 14/003 du 11 février 2014 relative à la conservation de la nature	Framework for the conservation of the biological diversity, the sustainable use of natural resources and the fair sharing of benefits arising from the use of biological and genetic resources.	https://www.fao.org/faolex/results/details/en/c/LEX-FAOC140376
DRC	Décret n° 14/019 du 02 août 2014 fixant les règles de fonctionnement des mécanismes procéduraux de la protection de l'environnement	Guide for the evaluation of environmental impact, conducting environmental audits and other procedures of environmental protection.	https://www.fao.org/faolex/results/details/en/c/LEX-FAOC140363

DRC	Loi n° 15/026 du 31 décembre 2015 relative à l'eau	Rules for the equitable and sustainable use of all water resources.	https://www.fao.org/faolex/results/details/en/c/LEX-FAOC154965
Zambia	Noxious Weed Act 1953	Mandates for the eradication of noxious weeds.	https://www.parliament.gov.zm/node/1040
Zambia	Environmental Management Act 12 of 2011	Framework for integrated environmental management and the protection and conservation of the environment and the sustainable management and use of natural resources.	https://www.parliament.gov.zm/node/7348
Zambia	Forest Act 4 of 2015	Rules for the establishment, declaration and management of National Forests, Local Forests, joint forest management areas, botanical reserves, private forests and community forests, as well the implementation of related international frameworks and mechanisms.	https://www.parliament.gov.zm/node/4535
Zambia	The Zambia Wildlife	Framework for the management of protected areas, conservation and sustainable use of biodiversity and artefacts of historical and scientific importance and the establishment of the implementing mechanisms and authorities.	https://www.parliament.gov.zm/node/4533
Zambia	Plant Pests and Diseases Act	Mandates for the eradication and prevention of the spread of plant pests and diseases.	https://www.parliament.gov.zm/node/1046
Zambia	Water Resources Management Act 21 of 2011	Framework for the management, development, conservation, protection and preservation of the water resource and its ecosystem.	https://www.parliament.gov.zm/node/6544

Zimbabwe	Environmental Management Act 2002	Framework for the sustainable management of natural resources and protection of the environment.	https://www.fao.org/faolex/results/details/en/c/LEX-FAOC047834/
Zimbabwe	Forest Act 1949	Framework for the administration, management, protection and sustainable use of forests and the products of the forest.	https://www.fao.org/faolex/results/details/en/c/LEX-FAOC008943/
Zimbabwe	Forest Amendment Act 2021	Amendments to Forest Act 1949 to extend mandates.	https://www.fao.org/faolex/results/details/en/c/LEX-FAOC221566
Zimbabwe	Plant Pest and Diseases Act 1958	Mandates for eradication and prevention of the spread of plant pests and diseases.	https://www.fao.org/faolex/results/details/en/c/LEX-FAOC060741/
Zimbabwe	Parks and Wildlife Act 1975	Framework for the establishment, management and sustainable use of national parks, botanical reserves, botanical gardens, sanctuaries, safari areas and recreational parks and the biodiversity in them.	https://www.fao.org/faolex/results/details/en/c/LEX-FAOC008942/
Zimbabwe	Water Act 1958	Rules for the planning of the optimum development and utilization of the water resources.	https://www.fao.org/faolex/results/details/ru/c/LEX-FAOC001217/
